# An Adaptive and Hybrid End-Point/Joint Impedance Controller for Lower Limb Exoskeletons

**DOI:** 10.3389/frobt.2018.00104

**Published:** 2018-10-22

**Authors:** Serena Maggioni, Nils Reinert, Lars Lünenburger, Alejandro Melendez-Calderon

**Affiliations:** ^1^Department of Health Science and Technology, ETH Zürich, Zurich, Switzerland; ^2^Hocoma AG, Volketswil, Switzerland; ^3^Cereneo Advanced Rehabilitation Institute (CARINg), Vitznau, Switzerland; ^4^Department of Physical Medicine and Rehabilitation, Northwestern University, Chicago, IL, United States

**Keywords:** assist-as-needed, impedance, gait trainer, exoskeleton, stiffness, rehabilitation, Lokomat, adaptive control

## Abstract

Assist-as-needed (AAN) algorithms for the control of lower extremity rehabilitation robots can promote active participation of patients during training while adapting to their individual performances and impairments. The implementation of such controllers requires the adaptation of a control parameter (often the robot impedance) based on a performance (or error) metric. The choice of how an adaptive impedance controller is formulated implies different challenges and possibilities for controlling the patient's leg movement. In this paper, we analyze the characteristics and limitations of controllers defined in two commonly used formulations: joint and end-point space, exploring especially the implementation of an AAN algorithm. We propose then, as a proof-of-concept, an AAN impedance controller that combines the strengths of working in both spaces: a hybrid joint/end-point impedance controller. This approach gives the possibility to adapt the end-point stiffness in magnitude and direction in order to provide a support that targets the kinematic deviations of the end-point with the appropriate force vector. This controller was implemented on a two-link rehabilitation robot for gait training—the Lokomat®Pro V5 (Hocoma AG, Switzerland) and tested on 5 able-bodied subjects and 1 subject with Spinal Cord Injury. Our experiments show that the hybrid controller is a feasible approach for exoskeleton devices and that it could exploit the benefits of the end-point controller in shaping a desired end-point stiffness and those of the joint controller to promote the correct angular changes in the trajectories of the joints. The adaptation algorithm is able to adapt the end-point stiffness based on the subject's performance in different gait phases, i.e., the robot can render a higher stiffness selectively in the direction and gait phases where the subjects perform with larger kinematic errors. The proposed approach can potentially be generalized to other robotic applications for rehabilitation or assistive purposes.

## Introduction

Exoskeletons for gait rehabilitation or walking assistance in subjects with neurological injuries have flourished in the last decades (Esquenazi et al., 2017). These devices seek to control the leg segments of the user and try to restore a gait pattern that is both physiological (i.e., following kinematic characteristics observed in non-impaired individuals) and safe. An effective rehabilitation device should not only control the movements of a patient's legs, but should also challenge the patient and promote his active participation (Lotze et al., 2003; Hogan et al., 2006). One way to achieve the latter is through adaptation of the robotic support based on the user's capabilities (Cai et al., 2006; Marchal-Crespo and Reinkensmeyer, 2009). This concept is known as *Assist-As-Needed* (AAN) (Emken et al., 2005).

The simplest and most common method of modifying the level of robotic support is with impedance controllers, where impedance is defined as any dynamic operator that outputs a force (or a torque) from a kinematic input (e.g., displacement, velocity) (Hogan, 1985). In most available exoskeletons, the impedance parameters must be manually adapted by therapists based on their experience [e.g., Lokomat® (Hocoma AG, Switzerland) (Colombo et al., 2000), LOPES II (University of Twente, The Netherlands) (Meuleman et al., 2016), HAL (Cyberdyne Inc., Japan) (Nilsson et al., 2014)]. New controllers that automatically adapt the impedance of the joints based on the user's performance have been proposed (Emken et al., 2008; Koopman et al., 2013; Maggioni et al., 2015), but not extensively implemented due to safety requirements. The use of adaptive control algorithms increases the compliance of the exoskeleton to the user's movements. Too much compliance (i.e., a low mechanical impedance) can lead to unsafe conditions because support may not be provided against users' errors, which may lead to tripping and injuries. The challenge in implementing adaptive controllers in lower limb exoskeletons is to find an appropriate trade-off between compliance (i.e., freedom of movement) and safety.

The choice of how an adaptive impedance controller is formulated inevitably determines how complex it is to address any potential hazard situation arising from reduced impedance. Here, we analyze the characteristics and limitations of controllers defined in two commonly used formulations: joint and end-point space, exploring especially the implementation of AAN controllers in these two spaces. A comparative analysis of these two approaches has been reported for industrial manipulators (Smith et al., 2014, 2015) but, to the best of our knowledge, such comparison has not been extensively examined within the context of rehabilitation robotics and even less in *lower limb* applications.

After analyzing the properties of these two approaches for the control of lower limb exoskeletons, we propose an AAN impedance controller that combines the strengths of working in both spaces: a hybrid joint/end-point impedance controller. This controller gives the possibility to adapt the end-point stiffness in magnitude and direction, to provide a support that targets end-point deviations with the appropriate force vector. This controller was implemented and tested on a two-link rehabilitation robot for gait training with actuated hip and knee joints—the Lokomat®Pro v5 (Hocoma AG, Switzerland). We present the proof-of-concept for this hybrid controller based on simulations and tests conducted with five able-bodied subjects and one subject with walking impairment due to a complete spinal cord injury. The proposed approach can potentially be generalized to other robotic applications for rehabilitation or assistive purposes.

## Joint vs. end-point space formulations

### Background concepts

**Figure 1:**
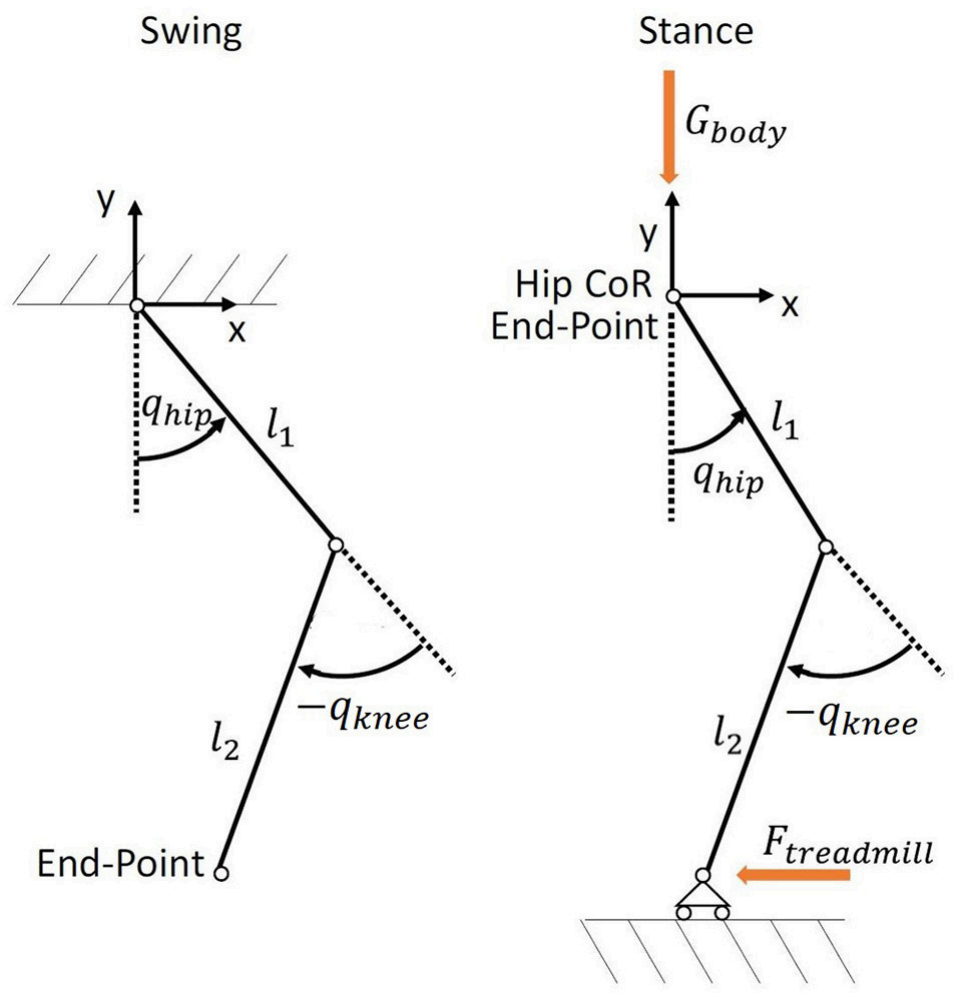
Comparison between the swing **(Left)** and stance **(Right)** models. During the swing phase, the ankle constitutes the end-point of the kinematic chain. During the stance phase, the end-point is the hip CoR. The force generated by the treadmill acts at the ankle, whose position is constrained in the vertical direction by the treadmill.

In this paper we analyze the implications of using joint or end-point space formulation for the control of lower limb exoskeletons. We model these systems as two-segment exoskeletons with a shank and a thigh segment. In the swing phase, the system can be modeled as a two-segment pendulum: the upper segment is fixed to the hip center of rotation (CoR) and the end-point corresponds to the ankle position (Kuo and Donelan, 2010). In the stance phase, the model is an inverse two-segment pendulum: after heel contact, the foot can only be moved backwards by the treadmill, hence the end-point of the kinematic chain is the hip CoR and not the ankle joint (Figure 1).

To analyze the impedance properties of the joint and end-point control approaches for the control of lower limb exoskeletons, we present the impact that these two formulations have on the end-point stiffness. The stiffness can be visualized as an ellipse, whose major axis indicates the direction of maximum stiffness (Mussa-Ivaldi et al., 1985; Shadmehr, 1993). The stiffness ellipse captures the geometrical features of the force field around a reference position of the end-point. In the force field representation, we can visualize the direction and magnitude of the restoring forces for displacements around the reference trajectory. For further details on the calculation of stiffness ellipses and force field, see the Appendix 1.

### Impedance control based on joint space formulation

In most exoskeleton devices, the actuators control the flexion and extension of the robotic joints, which roughly align to the human joints. Therefore, it is common to implement impedance controllers that compute the actuators' torques in order to follow reference trajectories defined in joint space (e.g., hip and knee angles). Furthermore, instrumented gait analysis increased our familiarity with angular kinematics and kinetics of the human joints.

A joint controller can be applied both in the stance and the swing phase of gait, because the actual joint trajectory **q**_*act*_ and the reference trajectory **q**_*ref*_ are defined continuously during the whole gait cycle and do not depend on the kinematic configuration (e.g., open chain in swing phase or closed chain in double-support phase). A joint space formulation avoids problems that might arise from inverse kinematics/dynamics calculations, especially in kinematic configurations (specific combinations of hip/knee angles) where the Jacobian matrix is singular.

For a two-link exoskeleton robot, the *joint* reference trajectory can be expressed as **q**_*ref*_ = [*q*_*hip*_, *q*_*knee*_], while **q**_*act*_ refers to the measured angles while the subject is walking. The torques τ_**q**_ to control the robotic actuators are provided by a motion controller with stiffness **K**_*q*_ = [*K*_*hip*_ 0; 0 *K*_*knee*_] and damping **B**_*q*_ = [*B*_*hip*_ 0; 0 *B*_*knee*_] (Equation 1).

(1) $$\begin{array}{l} \tau_{q}={\text{K}}_{q}\left({\text{q}}_{ref}-{\text{q}}_{act}\right)+{\text{B}}_{q}\left({\overset{\cdot }{\text{q}}}_{ref}-{\overset{\cdot }{\text{q}}}_{act}\right) \end{array}$$

Generally, in addition to the control torques τ_**q**_, robotic exoskeletons have a separate component τ_*comp*_, which compensates the inherent robot dynamics such as gravity, friction or inertia (e.g., Riener et al., 2005; Vallery et al., 2009).

#### Selection of joint reference trajectories

Joint reference trajectories ***q***_*ref*_ can be taken from literature (e.g., Winter, 1991; Perry, 1992; Stoquart et al., 2008), or from recordings of able-bodied subjects walking “freely” (i.e., in “transparent mode,” where only τ_*comp*_, but not τ_*q*_, is applied) in the same device to be controlled (Colombo et al., 2000). When determining ***q***_*ref*_, attention must be paid to avoid unwanted contact between the end-point (e.g., the heel or the tip of the foot) and the ground. For example, a small angular deviation at the knee joint may result in a considerable change in foot clearance (Winter, 1992).

One challenge in joint space formulation comes with the high inter-subject variability in angular patterns, which makes it difficult to define joint reference trajectories that fit all subjects. In some exoskeletons, ***q***_*ref*_ can be changed manually by the user within some limits (Riener et al., 2010; Meuleman et al., 2016). However, it is difficult to predict whether the subject will have adequate foot clearance and step length, since these also depend on the length of the thigh and shank segments.

Another challenge comes in applications where the users are required to perform a task following visual feedback, e.g., to follow a reference trajectory displayed on the screen. Simultaneous feedback from two or more joint space variables (e.g., hip and knee) is usually quite complex to process (Maggioni et al., 2015).

#### Impact of joint space formulation on end-point stiffness

Potential hazards during walking can come from unwanted interactions between the foot and the floor (or treadmill). Therefore, we examined the forces at the ankle level that may result in such unwanted interactions. These forces were generated by a controller defined in joint space, given foot displacements of different amplitude and directions throughout the swing phase. We obtained the resulting end-point forces (force field) using the Jacobian matrix of the two-links robot (see Appendix 1.2).

**Figure 2:**
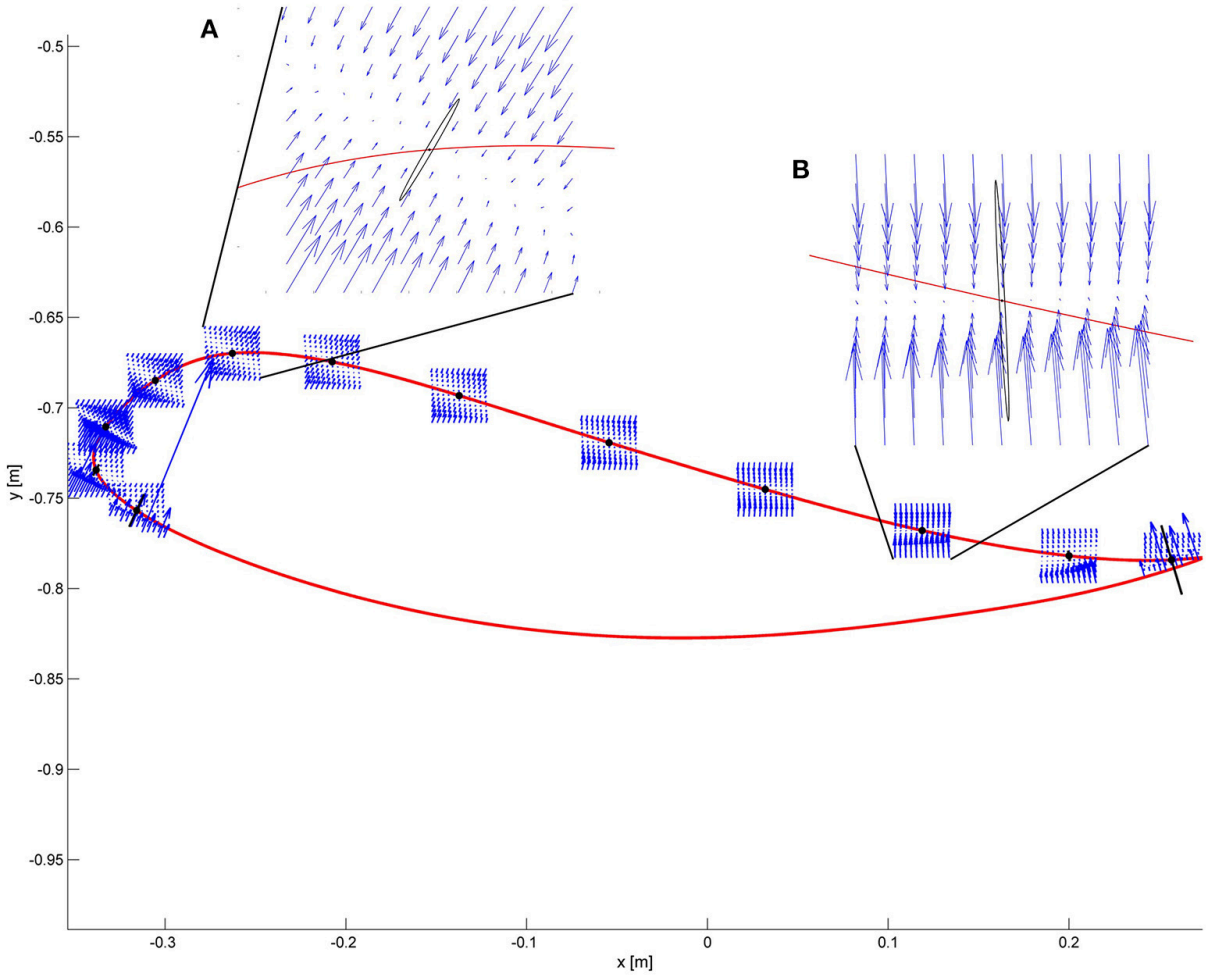
The force field resulting from a joint space impedance controller is shown at some selected points along the ankle trajectory. The restoring forces do not always point toward the reference position. Two critical points are magnified. **(A)** Point of maximum foot clearance: the vectors show that enough support is guaranteed if the ankle is below the reference trajectory. The ellipse in black represents the end-point stiffness resulting from the joint stiffness. **(B)** At the end of the swing phase, if the subject is late with respect to the reference point, it can experience forces directed downwards instead of forward.

**Figure 3:**
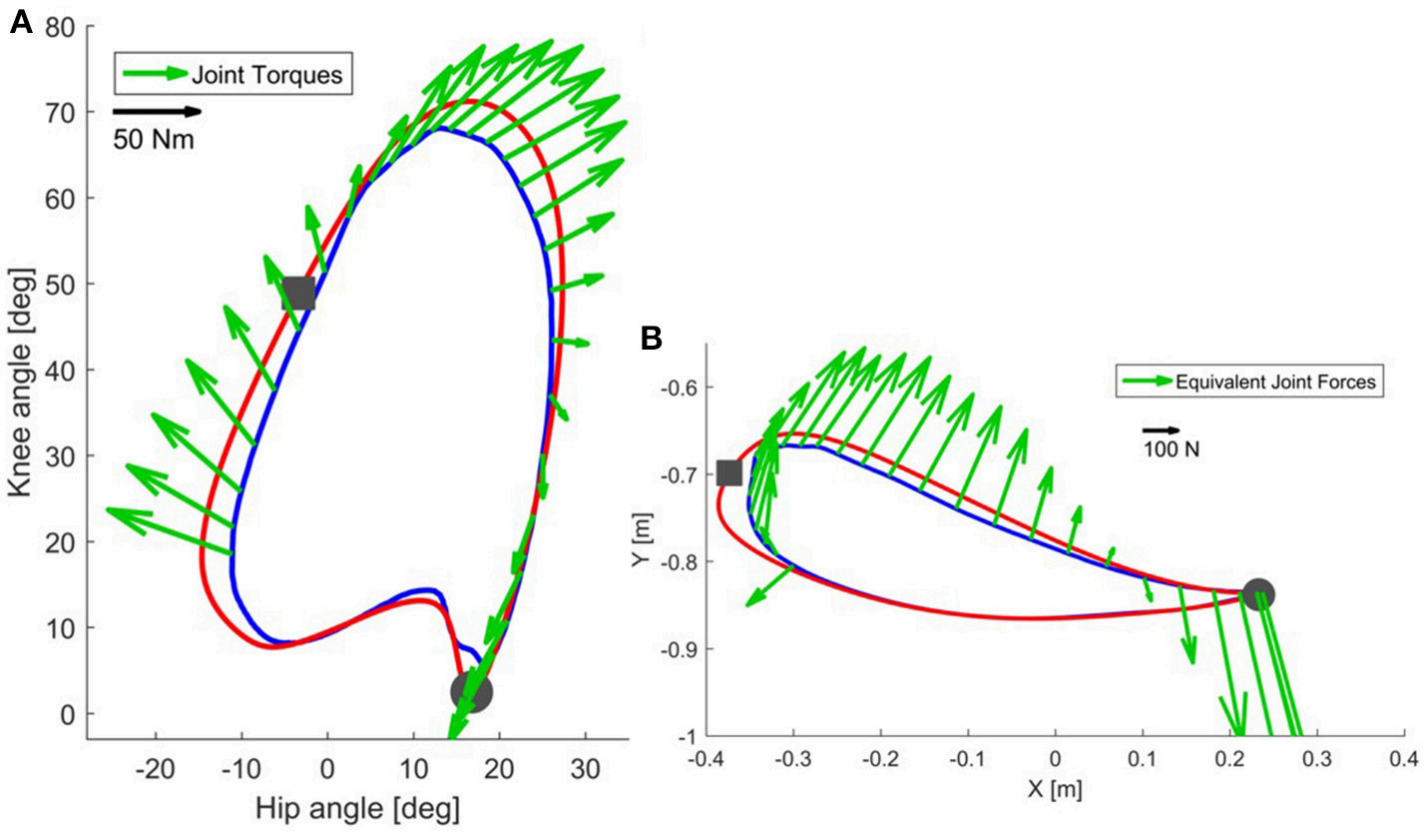
In correspondence of a real trajectory (blue line) deviating from the reference trajectory (red line), the joint controller generates the torques shown in **(A)**. The same torques can be visualized in end-point space **(B)** as equivalent end-point forces [see Appendix 1.4]. Refer to the scale for information on the magnitude of the torques and forces. The beginning of the stance phase is marked with a gray circle, while the beginning of the swing phase is marked with a gray square.

In Figure 2, we show the force field for different points during the pre-swing and swing phase. In this case, hip and knee stiffness are constant throughout the gait cycle, but the resulting end-point stiffness varies depending on the angular configuration of the joints. The magnitude and direction of joint torques and end-point forces applied by a joint controller on a real trajectory are presented in Figure 3. Two main requirements for functional walking are adequate foot clearance and foot placement at the end of swing (Gage, 1991; Baker, 2013). Therefore, we examined these two phases in detail. As the reader can appreciate, the restoring forces around the foot are not always directed toward the reference trajectory (note that the reference trajectory is defined in joint space, but it is transformed to end-point space for visualization purposes). Consider the situation where a subject is not able to sufficiently lift the foot from the ground at the beginning of swing phase: as we can see in Figures 2A, 3B, the joint controller is able to provide forces that are directed toward an adequate foot clearance position. On the other hand, if the subject is not able to perform a sufficiently long step (e.g., due to insufficient hip flexion or reduced knee extension at the end of swing), or if his foot is lagging behind the reference position, the actual position of his ankle can fall in an area where the forces rendered by the controller direct the foot toward the ground, instead of lifting it to guarantee a sufficient step length. It is interesting to compare how the same controller acts in the two different spaces; we can obtain insights that are not possible by studying the joint torques and end-point forces in isolation.

### Impedance control based on end-point space formulation

An alternative option to a joint space formulation is an *end-point space formulation* (sometimes referred to as *task space formulation*), in which the reference trajectory is defined according to an anatomical landmark around an end-point. In walking, the definition of end-point depends on the kinematic configuration, e.g., lateral malleolus or foot metatarsal during swing phase; or trochanter during stance phase, as the foot is already placed on the ground. Thus, formulating the problem in end-point space for lower limb exoskeletons may require two different control approaches: one for stance and another one for swing. While the implementation of this approach may be cumbersome in practice, a controller during swing that relies on an end-point space formulation may provide additional benefits compared to a joint space approach. In this paper, we are interested in studying the control of the end-point impedance only in the swing phase of gait.

In an end-point space formulation, the torque applied to the exoskeleton actuators is derived from an end-point force **F**_*x*_ (Equation 2). This force depends on a set of stiffness, **K**_**x**_ = [*K*_*xx*_*, K*_*xy*_*; K*_*yx*_*, K*_*yy*_], and damping, **B**_**x**_ = [*B*_*xx*_*, B*_*xy*_*; B*_*yx*_*, B*_*yy*_], parameters and a kinematic error between a measured end-point trajectory, **x**_*act*_ = [*x*_*act*_ *y*_*act*_], and a reference trajectory, **x**_*ref*_ = [*x*_*ref*_ *y*_*ref*_]. Note that *x*_*ref*_ and *x*_*act*_ can be calculated in real-time by using forward kinematic equations that depend on the measured joint angles *q*_*ref*_ and *q*_*act*_ and known limb segment lengths of the user (see Appendix 1.1). The accuracy of this calculation, however, depends on the correct measurement of the segments' lengths and on the alignment between the robotic joints and the human joints.

(2) $$\begin{array}{l} {\text{F}}_{x}={\text{K}}_{x}\left({\text{x}}_{ref}-{\text{x}}_{act}\right)\;+\;{\text{B}}_{x}\left({\overset{\cdot }{\text{x}}}_{ref}-{\overset{\cdot }{\text{x}}}_{act}\right) \end{array}$$

Using the Jacobian matrix **J**(**q**_*act*_), we obtain through inverse dynamics the torque that the joint actuators need to render the force **F**_*x*_:

(3) $$\begin{array}{l} \tau_{x}=\text{J}{\left[{\text{q}}_{act}\right]}^{T}{\text{F}}_{x} \end{array}$$

#### Selection of end-point reference trajectories

In contrast to joint reference trajectories, end-point trajectories are not widely available in the literature. One could take joint reference trajectories and apply forward kinematics, or obtain such trajectories experimentally. Another approach is to take a few features that ensure that the position of the foot guarantees a safe interaction with the environment, e.g., foot clearance and step length. These features can be easily visualized and adapted in end-point space. The manual adaptation of **x**_*ref*_ is more intuitive for therapists if they reason in end-point space (Emken et al., 2008) and focus on specific gait subtasks (Meuleman et al., 2016), rather than setting hip and knee angular reference trajectories simultaneously.

The subject can be provided with visual feedback regarding the position of his foot and asked to control its trajectory, in a similar way he is required to do in real environments—e.g., by lifting a foot over an obstacle. In gait trainer device literature, similar approaches have been followed when the focus was on ankle height to guarantee appropriate foot clearance in stiff-knee gait (Koopman et al., 2013). Additionally, visual feedback containing information about the end-point is much easier to process (Banala et al., 2009; Koopman et al., 2013; Krishnan et al., 2013) for subjects, whereas it is extremely difficult to adapt behavior based on feedback about hip and knee movements (Maggioni et al., 2015).

#### Impact of end-point space formulation on end-point stiffness

**Figure 4:**
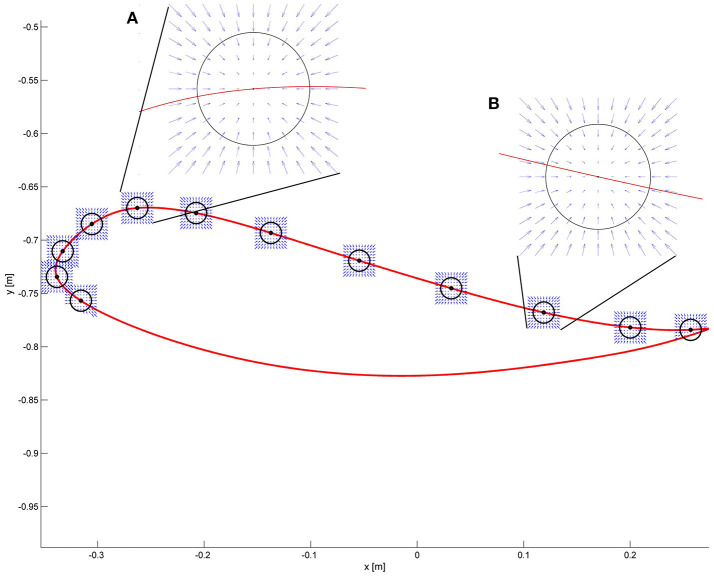
The desired force field in task-space is shown at some selected points along the end-point trajectory. The force field always points toward the reference position. Two critical areas are magnified. **(A)** Point of maximum foot clearance: the circle in black represents the desired end-point stiffness. The arrows show that regardless of the deviation from the reference point, the restoring force results always in a force directed to the reference point. **(B)** At the end of the swing phase, the desired characteristics of the force field are the same as in **(A)**.

Similar to section Impact of Joint Space Formulation on End-Point Stiffness, we would like now to examine the forces acting at the level of the foot when end-point control is used. By design (Equation 2), at each point of the swing phase, the restoring force for every deviation in Cartesian space is directed toward the reference end-point position (Figure 4), which is the point that could have potentially critical collisions with the environment (e.g., stumbling). The axes of the stiffness ellipse can be modified in magnitude and direction as desired. For example, a higher stiffness in the direction of gravity can be designed. However, singularities exist which prevent the end-point controller from generating joint torques in correspondence of those points (i.e., when the knee is completely extended at the end of swing).

**Figure 5:**
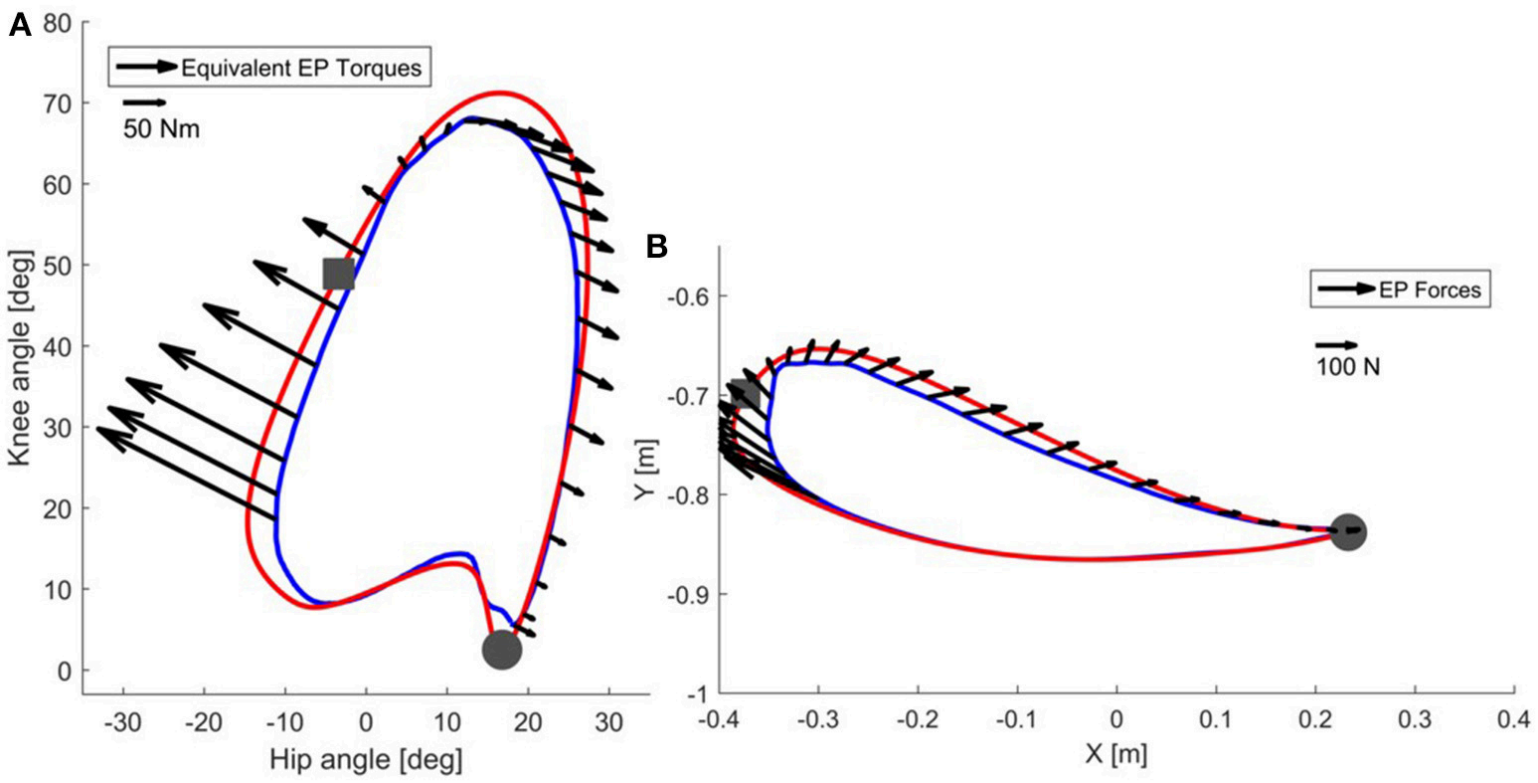
In correspondence of a real trajectory (blue line) deviating from the reference trajectory (red line), the end-point controller generates the forces shown in **(B)**. The same forces can be visualized in joint space **(A)** as equivalent joint torques [see Equation (3)]. Refer to the scale for information on the magnitude of the torques and forces. The beginning of the stance phase is marked with a gray circle, while the beginning of the swing phase is marked with a gray square.

Now consider the end-point forces generated when an end-point controller is used with a real trajectory. The force field set as shown in Figure 4 leads, in the case of the real trajectory presented in Figure 5B, to forces directed toward the reference trajectory in end-point space. Figure 5A shows the same forces transformed to torques Equation (3). As visible in the graph, the joint torques in this case do not always point toward the joint reference trajectory, especially at initial swing, the phase that is crucial for determining a safe foot clearance through an appropriate knee flexion. When the foot is lagging behind the reference trajectory in end-point space, the end-point controller tries to push the foot forward by increasing the hip flexion, while not acting on the knee. This is evident in Figure 5A where, at the point of maximum knee flexion, the torques have an almost null component acting on the knee. This problem might cause insufficient foot clearance and potential undesired foot contact with the treadmill.

### Assist-as-needed controllers

#### General formulation

“Assist-As-Needed” (AAN) refers to a control strategy based on assisting the patient/user only as much as needed to successfully perform a predefined task (Emken et al., 2005). One way to modulate the assistance provided by the robotic device is to modify the mechanical impedance rendered by the exoskeleton. A common AAN algorithm for an impedance controller typically updates a normalized impedance parameter P (P∈ℝ| 0≤P≤1), e.g., stiffness or damping, at every gait step *s*:

(4) $$P_{s+1}=\gamma \;P_{s}+f\left(e_{s}\right)\;g$$

A forgetting factor, γ(γ∈ℝ| 0<γ<1), limits the excessive reliance on the robotic assistance provided by the motion controller (the “slacking” effect; Marchal-Crespo and Reinkensmeyer, 2009). A gain g(g∈ℝ| g>0) adjusts the control parameter according to an error function $f\left(e_{s}\right),f(f:e_{s}\to [0,1])$, where *e*_*s*_ can be, for example, the kinematic deviation between the reference and actual trajectory of an exoskeleton. The function *f* may account for physiological kinematic variability (e.g., by defining a “deadband” around the reference trajectory; Banala et al., 2007; Emken et al., 2007). Note that domain of the parameters *P*, γ and *f*(*e*_*s*_) can be different, depending on the behavior one would like to achieve with the AAN algorithm (Marchal-Crespo and Reinkensmeyer, 2009), however, for the examples discussed further in this paper we have selected the ones above.

#### Joint space formulation of an AAN controller

There are several examples of controllers that adapt the robotic joint impedance of an exoskeleton to the subject's ability to walk - for a review see: (Marchal-Crespo and Reinkensmeyer, 2009; Hussain et al., 2011; Cao et al., 2014). For example, to create a patient-cooperative strategy for the Lokomat, hip and knee impedances were adapted according to the patient's effort (as estimated by the robot force sensors) (Riener et al., 2005). Based on a similar estimation of the subject's active contribution, Hussain adapted the joint impedance of a pneumatic-actuated exoskeleton robot (Hussain et al., 2013). However, both works were based on forces exerted by a limited group of able-bodied subjects, which could heavily compromise their applicability in patients exhibiting clonus or spasticity. In the Lokomat, this dependence on the interaction forces was overcome by implementing an approach called “Path control,” which allows freedom of movement around predefined joint trajectories, while a virtual tunnel of adjustable width guarantees safety (Duschau-Wicke et al., 2010).

In Maggioni et al. (2015), we presented an AAN algorithm that automatically adapts the Lokomat actuators' impedance based on the ability of the subject to follow a reference gait trajectory. In this work, the algorithm described by Equation (4) was applied. The control parameters **P** were the stiffness **K** and the damping **B** of the hip and knee in an impedance joint controller. The estimator of the subject's performance relied on the kinematic deviation between the actual trajectory and the reference. The gait cycle was divided in 30 windows. For each window *w* and for each step *s* the joint impedance was defined by one set of parameters, **K**_*s, w*_ and **B**_*s, w*_, which was adapted according to the weighted kinematic error performed in each window and every step.

(5) $$\begin{array}{l} {\text{K}}_{s+1,w}=\gamma_{1}{\text{K}}_{s,w}+g_{1}{f_{1}\left[e_{s}\right]}_{w} \end{array}$$

(6) $$\begin{array}{l} {\text{B}}_{s+1,w}=\gamma_{2}{\text{B}}_{s,w}+g_{2}{f_{2}\left[\overset{\cdot }{{\text{e}}_{s}}\right]}_{w} \end{array}$$

**Figure 6:**
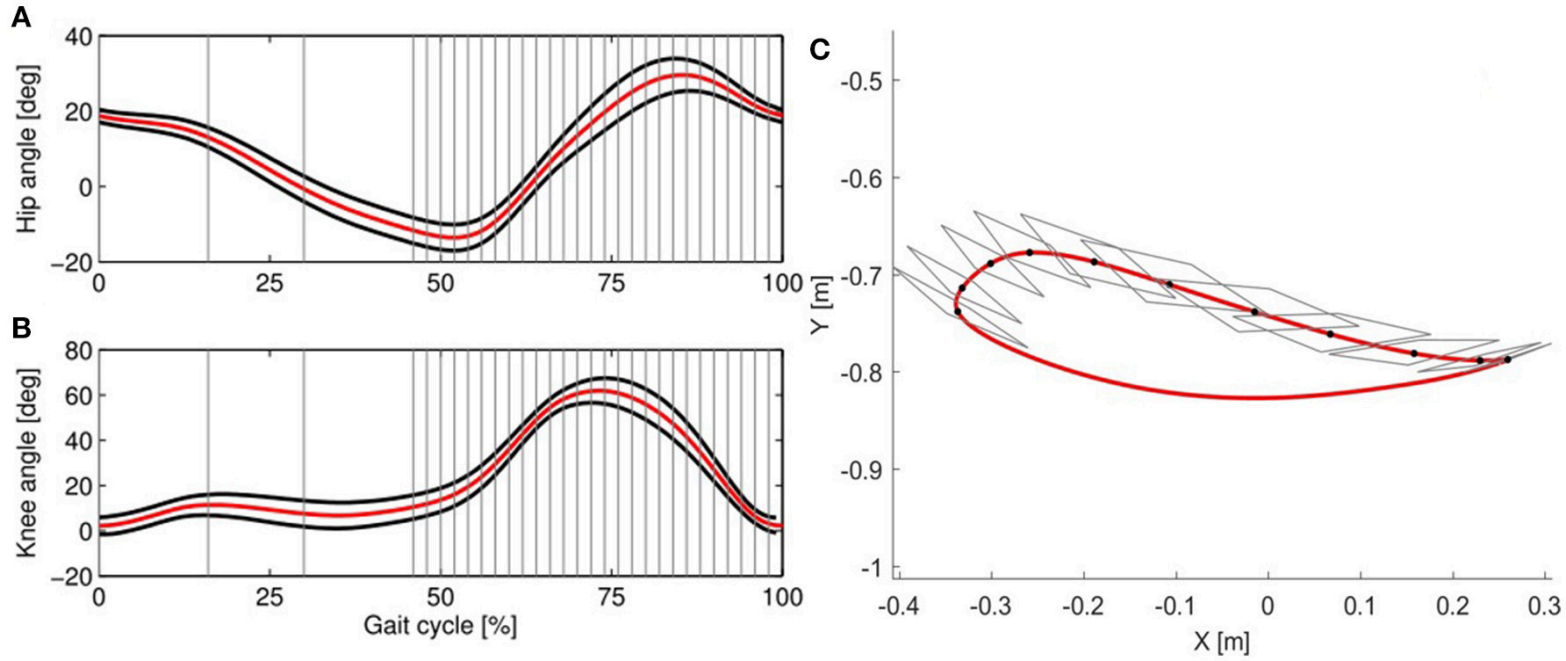
In the joint controller hip and knee deadbands are defined independently from each other, as shown in the **(A)** (hip angle) and **(B)** (knee angle). The reference trajectory (red) is taken from Colombo et al. (2000). The deadbands (black lines) are calculated from the standard deviation of the trajectories of 10 able-bodied subjects walking in the Lokomat with impedance set to 5% of the maximum, which allows freedom of movement. In the AAN algorithm, deviations occurring within the deadbands lead to a null error. The gait cycle is divided in 30 windows (gray lines show the windows' limits). In **(C)**, the resulting reference trajectory (red) and the corresponding deadbands in end-point space are shown. For each window along the swing phase (only 15 are shown for clarity of representation), the gray rhomboid shows the area including all the possible combinations of hip and knee angles within the deadbands shown in **(A,B)**.

A set of gains γ_1_, γ_2_, *g*_1_, *g*_2_ were defined in order to have the impedance decrease slowly in the presence of physiological deviations and to react fast enough in case of large errors. The error weighting function *f* [**e**_*s*_]_*w*_ consisted of a hyperbolic tangent function of the kinematic error *e*_*s*_ defined for each window *w*, which allowed physiological deviations from the reference trajectories of the hip and knee joint, while ensuring safety. This means that for each time point of the gait cycle, the subject's hip and knee was allowed to deviate from the reference trajectory within the deadbands defined for each joint, independently from each other and irrespective of the position of the end-point. Suitable deadbands in joint-space can be defined based on normal ranges for hip and knee joint angles (e.g., taking normative data from Winter, 1991; Perry, 1992; Stoquart et al., 2008 or from able-bodied people walking in the device). To study how these angular boundaries result in end-point space, we applied forward kinematics (see Appendix 1.1) to render the resulting boundaries around the end-point (i.e., at the ankle), as illustrated in Figure 6.

Due to the non-linearity of the kinematic transformation and its dependency on the joint configuration, the shape of the boundaries resulting at the end-point is hardly predictable from what can be seen in joint space. During the push-off phase and at the beginning of swing, the boundaries are extremely narrow along the direction of the foot motion. This results in a very strict timing requirement for the subject walking in the robot (i.e., the subject must closely follow the desired ankle position at any time). Even small deviations along the directions of motion can result in a high error, which causes the algorithm to increase the impedance in this specific gait phase. However, in the direction perpendicular to the reference trajectory, higher deviations are allowed, and they could potentially result in insufficient foot clearance. Conversely, during mid-swing, the resulting shape of the joint space deadbands is less conservative along the direction of the trajectory, allowing increased leading or lagging of the foot with respect to a reference position. At the end of swing, again the shape of the boundaries in end-point space changes: here the boundaries allow the subject to perform longer or shorter steps than desired.

#### End-point space formulation of an AAN controller

In this type of controller, the parameters **P** adapted based on Equation (4) are the end-point stiffness and damping (**K**_*x*_ and **B**_*x*_). In literature, there are several examples of end-point impedance adaptation implemented in exoskeleton and end-effector devices. Among the latter, Emken et al. adapted the end-point impedance of a robot guiding the ankle of the subject (ARTHuR) based on the position and velocity error between the reference and actual ankle trajectories (Emken et al., 2008). Hussein et al. implemented an algorithm for adapting the width of a deadband for velocity deviations in the footplate-based Gait Trainer GT-I (Reha-Stim, Germany): based on the error between actual and desired end-effector velocity; the deadband width was either increased to allow more freedom or decreased to provide more guidance to the subject (Hussein et al., 2009). Other works instead, despite using exoskeleton devices, developed an algorithm that adapted the end-effector impedance or force field and calculated the required joint torques based on end-point information. For example, Koopman et al. developed an adaptive vertical force acting on the ankle to support foot clearance (LOPES, Koopman et al., 2013); Banala et al. designed a force field acting on the ankle to guide the end-point along a virtual tunnel (ALEX, Banala et al., 2007).

Having control over the task space impedance allows the implementation of AAN controllers that provide optimal assistance to the end-point. Indeed, the task space force field can be shaped in order to support the foot only in the directions that are needed. Furthermore, designing the deadbands in end-point space allows requirements such as minimum foot clearance or minimum step length to be set directly.

### Summary of working in different spaces

The two controllers show very different features when applied to a two-link exoskeleton and it is not possible to prefer one over the other independently of the application. In Table 1, we summarized the strengths and weaknesses of the two control formulations. The symbols “+” and “–” indicate whether the formulation can adequately address the specific features listed. These aspects have also been nicely addressed in Smith et al. (2015), where the performance of joint and end-point controllers is compared in an industrial manipulator.

**Table 1:** Summary of the performances of joint and end-point formulations for the control of a two-link exoskeleton.

**Features**	**Joint space formulation**	**End-point space formulation**
Application in stance and swing phase	+	–
Intuitive definition of foot clearance and foot placement as safety parameters	–	+
Intuitive definition of deadbands for an ANN controller	–	+
Directional adaptation of end-point stiffness to provide adequate foot guidance	–	+
Intuitive control of robot (e.g., no need of inverse kinematic calculations)	+	–
Easiness of dealing with singular kinematic configurations	+	–

## Hybrid joint/end-point space controller with assist-as-needed

In section Joint vs. End-Point Space Formulations, we highlighted strengths and weaknesses of the two formulations: joint and end-point space. Here, we propose an adaptive controller that is formulated in both spaces (“hybrid” formulation) and aims to combine the strengths of both approaches. An end-point space component aims at adapting the end-point stiffness in both magnitude and direction to provide a guided foot placement; while a joint space component aims at providing appropriate temporal coordination between hip and knee angles, especially when the kinematic configuration of the exoskeleton is close to a singularity. This hybrid approach also gives the possibility of defining deadbands more intuitively (based on foot position), which gives more control over the interactions with the environment.

**Figure 7:**
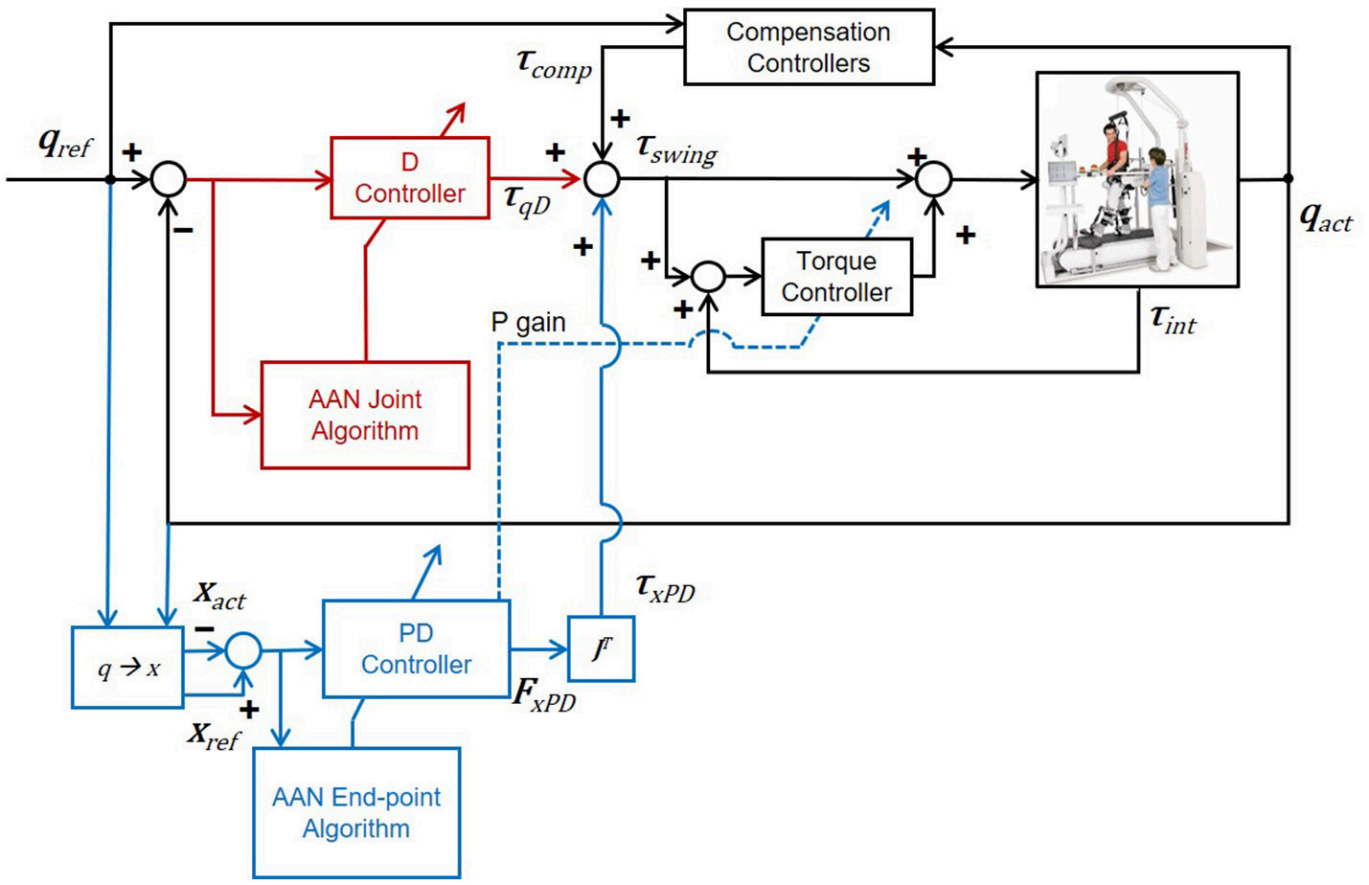
Control diagram of the adaptive hybrid joint/end-point controller during the swing phase of gait. The transparency of the exoskeleton is obtained through a torque feedback loop (torque controller). The torque controller provides a torque proportional to the error between the desired torque τ_*swing*_ and the measured torque τ_*int*_, in order to minimize this same error. Transparency is improved through the optimization of passive dynamics with a method called *Generalized Elasticities* (Vallery et al., 2009). Detailed information on the low-level control architecture can be found in Riener et al. (2005) and Vallery et al. (2009).

The torques applied during the swing phase of gait, τ_*swing*_, are the sum of torques generated by a PD controller based on the end-point position and end-point velocity error (Equation 8), torques generated by a D controller based on the angular velocity error in joint space (Equation 9), and compensation, as illustrated in Figure 7:

(7) $$\tau_{swing}=\tau_{xPD}+\tau_{qD}+\tau_{comp}$$

(8) $$\tau_{xPD}=J{\left[q_{act}\right]}^{T}K_{x}\left(x_{ref}-x_{act}\right)+\;J{\left[q_{act}\right]}^{T}B_{x}\left({\overset{\cdot }{x}}_{ref}-{\overset{\cdot }{x}}_{act}\right)$$

(9) $$\tau_{qD}=B_{q}({\overset{\cdot }{q}}_{ref}-{\overset{\cdot }{q}}_{act})$$

The end-point controller is designed to control the magnitude and direction of the forces required in task-space. Since the reference trajectories in joint space are derived from trajectories in task space, one can express the controller terms as:

(10) $$\begin{array}{lll} \tau_{xPD}+\tau_{qD} & = & {\text{K}}_{tot}\left({\text{q}}_{ref}-{\text{q}}_{\text{act}}\right)+{\text{B}}_{tot}\left({\overset{\cdot }{\text{q}}}_{ref}-{\overset{\cdot }{\text{q}}}_{act}\right) \end{array}$$

(11) $$\begin{array}{lll} {\text{K}}_{tot} & = & {\text{J}\left[q_{\text{act}}\right]}^{\text{T}}\;{\text{K}}_{x}\;\text{J}\left[q_{\text{act}}\right] \end{array}$$

(12) $$\begin{array}{lll} {\text{B}}_{tot} & = & {\text{J}\left[q_{\text{act}}\right]}^{T}{\text{B}}_{x}\;\text{J}\left[q_{\text{act}}\right]+{\text{B}}_{q} \end{array}$$

Note that the stiffness and damping matrices must fulfill the necessary conditions for stability defined in Appendix 2.

### AAN algorithm

The actual stiffness and damping in end-point space, **K**_*x*_[N/m] and **B**_*x*_[Ns/m], are obtained from a normalized stiffness and damping ${\bar{\text{K}}}_{x}\;$and ${\bar{\text{B}}}_{x}$ matrices, which are then scaled according to the specific characteristics of the robot. The normalized joint damping term ${\bar{\text{B}}}_{q}$ can be adapted according to Equation (6) in section Joint Space Formulation of an AAN Controller. ${\bar{\text{B}}}_{x}$ can be adapted either with a similar algorithm or coupled to ${\bar{\text{K}}}_{x}$.

For the term ${\bar{\text{K}}}_{x}$ we would like an AAN algorithm that adapts both the magnitude and direction of the equivalent stiffness ellipse based on the kinematic errors performed throughout the swing phase.

To achieve this the swing phase is divided into equally sized windows. For each window *w* and for each step *s*, we adapt the stiffness based on the weighted error at the previous step, both in magnitude and in direction, as:

(13) $${\bar{K}}_{x_{s+1,w}}=\;\gamma_{x}{\bar{K}}_{x_{s,w}}+f_{K_{x\;}}\left[e_{x_{s,w}}\right]\;R\left[\alpha_{s,w}\right]\;G_{K}\;R{\left[\alpha_{s,w}\right]}^{T}$$

(14) $$e_{x_{s,w}}=\;\left[\begin{array}{c} x_{ref_{s,w}}-x_{act_{s,w}}\\ y_{ref_{s,w}}-y_{act_{s,w}} \end{array}\right]$$

(15) $$\alpha_{s,w}=\arctan\left(e_{x_{s,w}}\right)$$

(16) $$R\left[\alpha_{s,w}\right]=\;\left[\begin{array}{c} \cos\alpha_{s,w}\;-\text{​}\sin\alpha_{s,w}\\ \sin\alpha_{s,w}\;\cos\alpha_{s,w} \end{array}\right]$$

**Figure 8:**
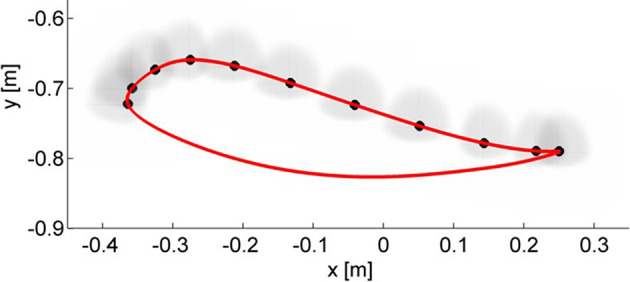
In end-point space the deadbands have been designed as asymmetric Generalized Gaussian functions (AGGF). The weighting functions are shaped differently in different points of the swing phase to prevent kinematic deviations that could result in unsafe interactions with the treadmill (e.g., reduced foot clearance and step length). For each window during the swing phase an AGGF is defined (for clarity of representation, only half of the windows are displayed). Kinematic errors falling within the borders of the respective AGGF result in a null weighted error. Otherwise, the weighted error saturates to 1.

The first term, $\gamma_{x}{\bar{\text{K}}}_{x_{s,w}}$, reduces the stiffness ellipse in all directions given a constant forgetting factor, γ_*x*_ = 0.9. The second term increases the stiffness in the direction of the kinematic error. The magnitude of this change is controlled by a gain matrix **G**_*K*_ = [0.1 0;0 0.01], which can be seen as a predefined ellipse with axes of fixed length. This ellipse **G**_*K*_ is (i) rotated along the direction of the error, (ii) scaled according to the magnitude of the weighted error *f*_*K*_*x*__ [**e**_*x*_*s, w*__] and (iii) summed to the stiffness ellipse $\gamma_{x}{\bar{\text{K}}}_{x_{s,w}}$. The error function *f*_*K*_*x*__ (*f*_*K*_*x*__ : **e**_*x*_*s, w*__ → [0, 1]) is defined for each window *w* with different shape characteristics (Figure 8). The error functions *f*_*K*_*x*__ [**e**_*x*_*s, w*__] can be defined with deadbands designed in end-point space. In this way, it is possible to identify requirements for the foot trajectory that ensure a safe interaction between the foot and the treadmill, for example, minimum foot clearance (Begg et al., 2007) and minimum step length (Sekiya et al., 1996). One way of defining the error weighting functions *f*_*K*_*x*__ [**e**_*x*_*s, w*__] is by using Asymmetric Generalized Gaussian functions (AGGF) (Elguebaly and Bouguila, 2013) which can be designed to have a different variance depending on the gait cycle window. The AGGF allows the width of tolerated kinematic deviations to be defined in all directions independently. An example is presented in Figure 8. By design, ${\bar{\text{K}}}_{x_{s,w}}\;$and *f*_*K*_*x*__ [**e**_*x*_*s, w*__] are bounded above by 1, therefore, even in presence of high errors, the eigenvalues of the stiffness matrix will never increase above the initial values. The change in the stiffness matrix between consecutive time steps can be bounded by the necessary stability conditions defined in the Appendix 2.

Due to the non-linear and adaptive nature of the controller (and the human) and to the variable impedance profile, it is a daunting task to derive the analytical necessary and sufficient conditions for stability. However, we believe that with the necessary (although not sufficient) conditions defined in the Appendix 2, in combination with a series of safety measures to prevent undesired robot behaviors, the safety of the user can be guaranteed. First and foremost, we made sure that the controller was stable with constant stiffness and damping values throughout the task space. Second, software mechanisms were in place to constrain the stiffness and damping values to the necessary boundaries defined in Appendix 2. The damping was tied to the stiffness to guarantee a critically damped (or overdamped) system throughout the different kinematic configurations. The rate of change of stiffness and damping parameters was constrained. Finally, the safety hardware and software mechanisms of the Lokomat prevented to reach singular configurations and shut down the motors whenever an excessive force or an excessive deviation from the reference trajectory was detected. Before the tests in humans, the controller was tested in real-life simulations on a test-bench as described in section Simulation Results.

## Simulation results

Before testing the AAN hybrid joint/end-point controller in human subjects, we performed simulations of the expected behavior using Matlab (v2013b, Mathworks).

We started from the simple case of a point along the reference trajectory and simulated different types of kinematic error. We wanted to test whether the AAN algorithm in the hybrid controller ensures an adaptation of the stiffness matrix to the direction and magnitude of the error. We simulated two cases: (i) error of unitary magnitude and constant direction (angle α between the error vector and the x axis equals 0) and (ii) error of unitary magnitude but variable direction (with α varying randomly at each step in the interval [0, π /2]). The resulting stiffness ellipses are described in terms of *size, shape*, and *orientation* (Mussa-Ivaldi et al., 1985), whereby *size* indicates the length of the major axis of the ellipse, *shape* the ratio between the major and minor axis of the ellipse, and *orientation* the angle between the major axis and the x axis.

**Figure 9:**
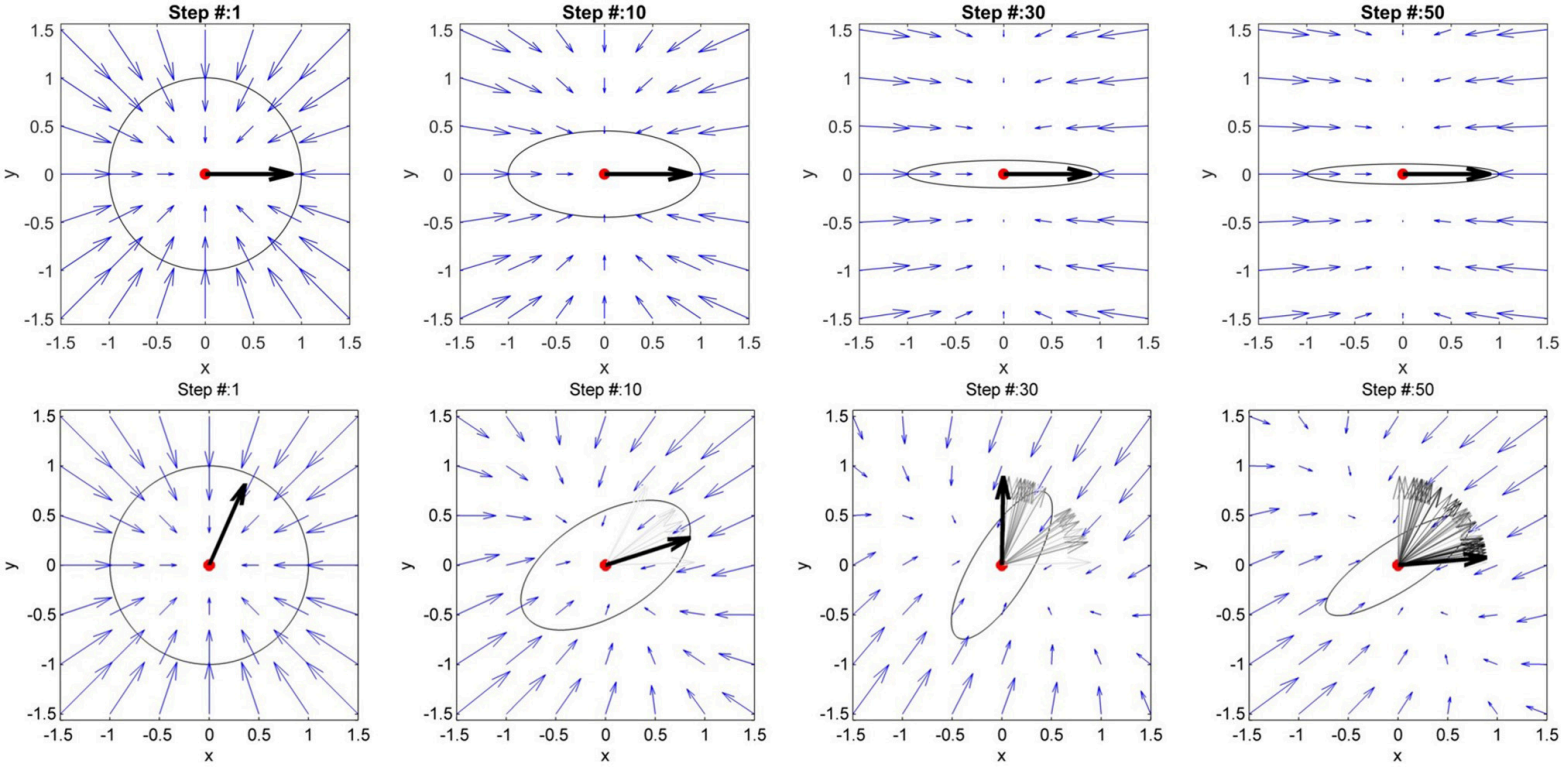
First line: simulation of an error with constant magnitude and direction (black vector) around a reference point (in red). The stiffness ellipse initial configuration is a circle which adapts step by step to the error. The central force field visible at step 1 consequently changes its characteristics. At step 50, the force field is directed mainly along the direction of the error. This implies that the stiffness is high only in directions parallel to the error. Second line: simulation of an error with constant magnitude and variable direction. The error angle variates randomly between 0 and 90°. The error of the current step is shown in bold black, while the previous vectors are shown in gray. The stiffness ellipse adapts its orientation based on the error direction. The force field represented by the blue vectors adapts accordingly.

In the first simulation (Figure 9—first line), the size along the error direction (length of the ellipse major axis) does not decrease since the error function *f*_*K*_*x*__ [**e**_*x*_*s, w*__] gives a constant unitary result (Equation 13). The orientation of the ellipse's major axis aligns with the error direction, inducing a force field with maximal restoring forces along the direction of the error and very low forces in every other direction, guaranteeing a compliant behavior of the controller against disturbances in directions other than the error. In the second simulation, as shown in Figure 9—second line, the ellipse orientation follows the error direction and so does the relative force field. The shape of the ellipse depends on how variable the direction of the error was in the previous steps (Equation 13).

**Figure 10:**
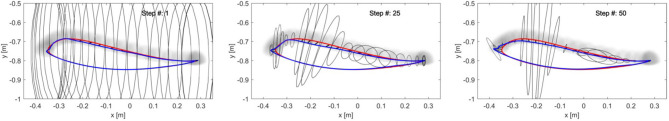
Adaptation of the end-point stiffness of the test orthosis during the simulation of a spastic-like behavior in the simulated human leg of the test bench. The initial stiffness ellipses are shown in the first box. The simulated velocity-dependent torque caused deviations of the foot trajectory at mid-swing and at the end of the swing phase. The hybrid controller adapted the stiffness ellipses magnitude and direction to provide targeted support to these deviations.

In a second phase, we used a robotic test bench to simulate neurological impairments such as spasticity. The test bench uses a bio-inspired model of a human leg implemented on the leg orthosis of a robotic gait trainer (the Lokomat, in this case). In this setup, one leg orthosis is controlled to simulate a human leg (*simulated human leg*), while the second orthosis (*test orthosis*) is controlled by the hybrid end-point/joint controller with AAN. The two orthoses are then rigidly connected using two aluminum bars, simulating a physical attachment of the robot to the user's leg. A spastic-like behavior was implemented on the simulated human leg by adding a velocity-dependent torque at the level of the knee joint, which was applied when the knee angular velocity exceeded a certain threshold. A detailed description of the test bench and of the impairment simulation can be found in Maggioni et al. (2016). The physical connection between the two orthoses allowed the hybrid controller implemented on the test orthosis to control the simulated human leg by shaping the stiffness ellipses to the simulated impairment. As expected, the test orthosis with the hybrid joint/end-point controller adapted the end-point stiffness to counteract the deviations of the simulated human leg caused by the spastic-like simulated impairment (Figure 10).

## Experimental results

The adaptive hybrid joint/end-point controller and the adaptive joint controller were tested with five able-bodied subjects (1 female, age = 27 ± 4.7 years) and one subject with a chronic motor complete Spinal Cord Injury (male, age = 37 years, ASIA B, level of injury = T4, WISCI II = 0/20). The Kantonale Ethikkommission Zürich and Swissmedic approved the study. The aim of this test was first to determine the feasibility and safety of the novel hybrid controller, and subsequently compare the performances of the adaptive hybrid controller to the existing joint adaptive controller (Maggioni et al., 2015). In particular, we hypothesized (i) that this novel controller adapts the magnitude of the stiffness to the subject's ability to follow the reference trajectory and, at the same time, (ii) that the orientation of the stiffness ellipses aligns to end-point deviations. We decided not to test the pure end-point controller on human subjects, due to safety concerns that emerged while doing preliminary tests with a dummy. As foreseen in section Impact of End-Point Space Formulation on End-Point Stiffness, the end-point controller alone was not able to guarantee sufficient foot clearance and avoid potential undesired foot contact with the treadmill.

### Methods

Subjects were instructed to follow a given foot trajectory in time and space, which was projected on a screen positioned in front of the Lokomat. The actual and reference ankle trajectories were displayed in different colors and two dots indicated the reference and actual position at every time point. After being set up in the Lokomat, the subjects were allowed to familiarize themselves with walking in the device with the standard impedance controller (impedance was set at the maximum available value). The visual feedback was constantly presented to the subject. In this familiarization phase, the Lokomat gait pattern was adjusted to the subject's gait pattern by tuning the ROM and the offset of the hip and knee angular trajectories. These settings were then kept constant during the subsequent experiment. Once comfortable and accustomed to walking inside the robot, the subject was presented with a familiarization round with the novel AAN hybrid controller as described in section Hybrid Joint/End-Point Space Controller With Assist-as-Needed. The subject was instructed to follow the reference trajectory as closely as possible while the adaptation algorithm adapted the impedance based on the kinematic error of the ankle trajectory. After the familiarization phase, the AAN control was active on the leg under test for 50 steps, while the impedance of the other leg was kept at the maximum available value. To ensure a safe foot clearance during swing, the stiffness in the vertical direction was made 5 times higher than the stiffness in the horizontal direction. While this is not a problem in the case of high impedance, it might become apparent when the adaptation algorithm reduces the impedance below a certain level, especially in patients with walking impairments. The implemented stiffness ${\tilde{\text{K}}}_{x}$ and damping ${\tilde{\text{B}}}_{x}$ in the Lokomat were:

(17) $$K_{x}=M_{K}{\bar{K}}_{x}$$

(18) $$M_{K}=\;\left[1500\;0;\;0\;7500\right]\frac{N}{m}$$

(19) $${\tilde{K}}_{x}=\frac{\left(K_{x}+K_{x}^{\;T}\right)}{2}$$

(20) $${\tilde{B}}_{x}=M_{B}{\bar{B}}_{x}$$

(21) $$M_{B}=\left[40\;0;0\;40\right]\frac{Ns}{m}$$

The transformation in Equation (19) guarantees that stiffness matrix is symmetric. In addition, to guarantee the stiffness matrix to remain positive definite after this transformation the following constraint was implemented:

(22) $$\begin{array}{l} \left(-\sqrt{K_{11}K_{22}}+\;\rho \right)<\;K_{ij}<\left(\sqrt{K_{11}K_{22}}-\;\rho \right) \end{array}$$

For *i* ≠ *j;i,j* = 1,2, where

(23) $$\begin{array}{l} \rho =0.1\;\sqrt{K_{11}K_{22}} \end{array}$$

The performance of the AAN hybrid controller was then compared with that of the AAN joint controller (see section Joint Space Formulation of an AAN Controller and Maggioni et al., 2015). For this comparison, subjects were tested in a separate session (scheduled within 4 weeks), while performing the same task using the AAN joint controller.

In the AAN hybrid controller, the magnitude of the end-point stiffness was calculated as the maximum eigenvalue of the stiffness matrix (i.e., the length of the major axis of the stiffness ellipse), averaged over all the windows during the swing phase of each step. The major axis of the stiffness ellipse indicates the direction where the end-point stiffness is maximal. To obtain a measure of the alignment between the direction of maximum stiffness and the position error at the ankle, we calculated the angle between the major axis of the stiffness ellipse and the vector of the end-point error. Only the swing phase of the gait is considered, since the hybrid controller is active only during swing. The weighted kinematic error *f*_*K*_*x*__ [**e**_*x*_*s, w*__] equals zero when the actual deviation is within the defined deadbands. In this case, the adaptation algorithm (Equation 13) decreases the size of the stiffness ellipse but does not change its orientation. Therefore, we only calculated the alignment in those windows where the weighted error *f*_*K*_*x*__ [**e**_*x*_*s, w*__] was greater than 0.1. The data of the last 5 steps of the adaptive task were used for the analysis of the final stiffness alignment determined by the algorithm. An average value over all subjects was calculated.

In the joint controller, the magnitude of the stiffness was calculated as the mean of the hip and knee joint stiffness during the swing phase. We then obtained the equivalent end-point stiffness resulting from the joint stiffness matrix (Equation A.10 in Appendix 1). The angle between the major axis of the resulting stiffness ellipse and the direction of the error in end-point space was calculated to estimate the alignment of the force field perceived at the ankle with the error.

### Results

All subjects were able to perform the experiment with the adaptive hybrid controller; the subject with SCI required a fixed body weight support equal to 70% of his body weight to use the adaptive hybrid controller.

The overall end-point stiffness decreased over time and converged to a specific value for each subject. The patient reached, as expected, a higher final value than the able-bodied subjects did.

**Figure 11:**
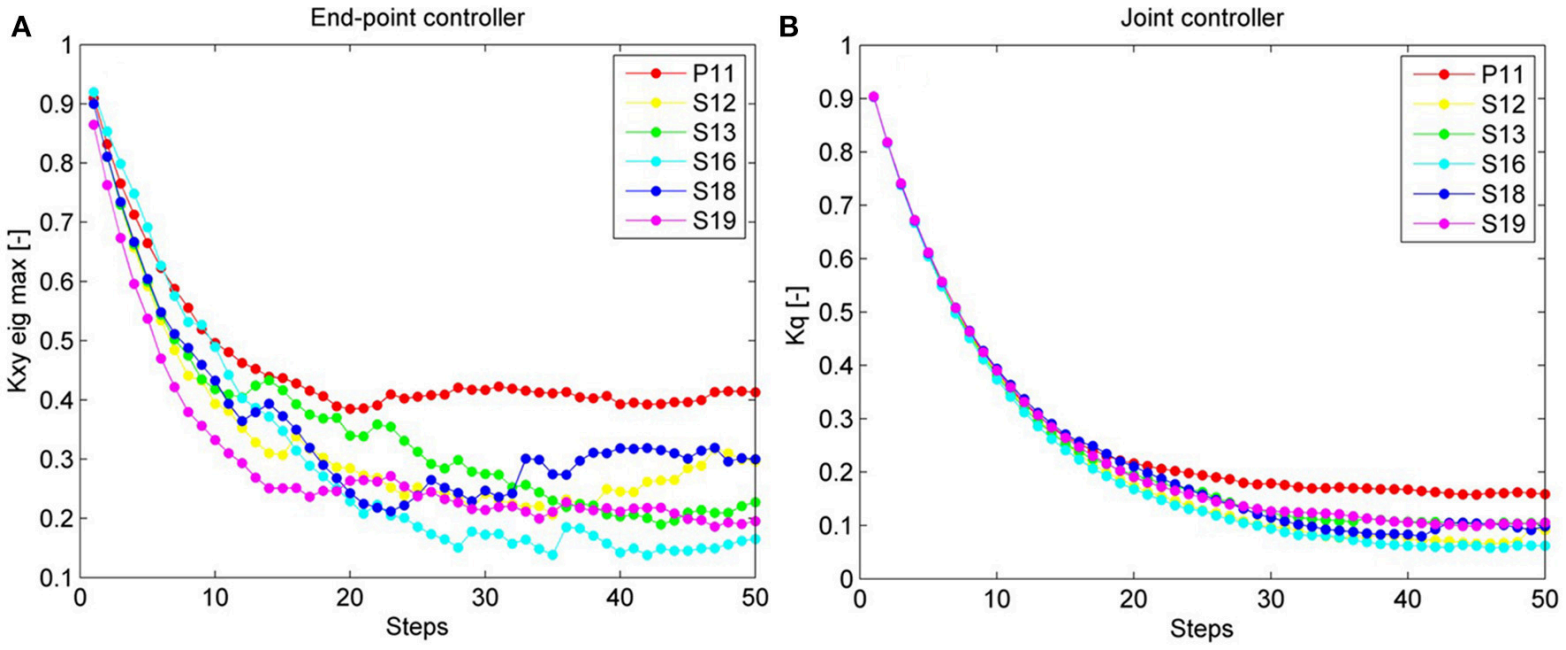
In this figure, the normalized adaptive stiffness of the two types of controller (AAN hybrid controller and AAN joint controller) is shown over 50 steps. Each data point represents the mean value over the swing phase of one step. In **(A)**, the adaptive stiffness of the hybrid controller (i.e., the maximum eigenvalue of the ellipse) is displayed. In **(B)**, the adaptive stiffness of the joint controller (i.e., the mean of the normalized hip and knee stiffness) is shown. The data of the patient are visualized in red.

**Figure 12:**
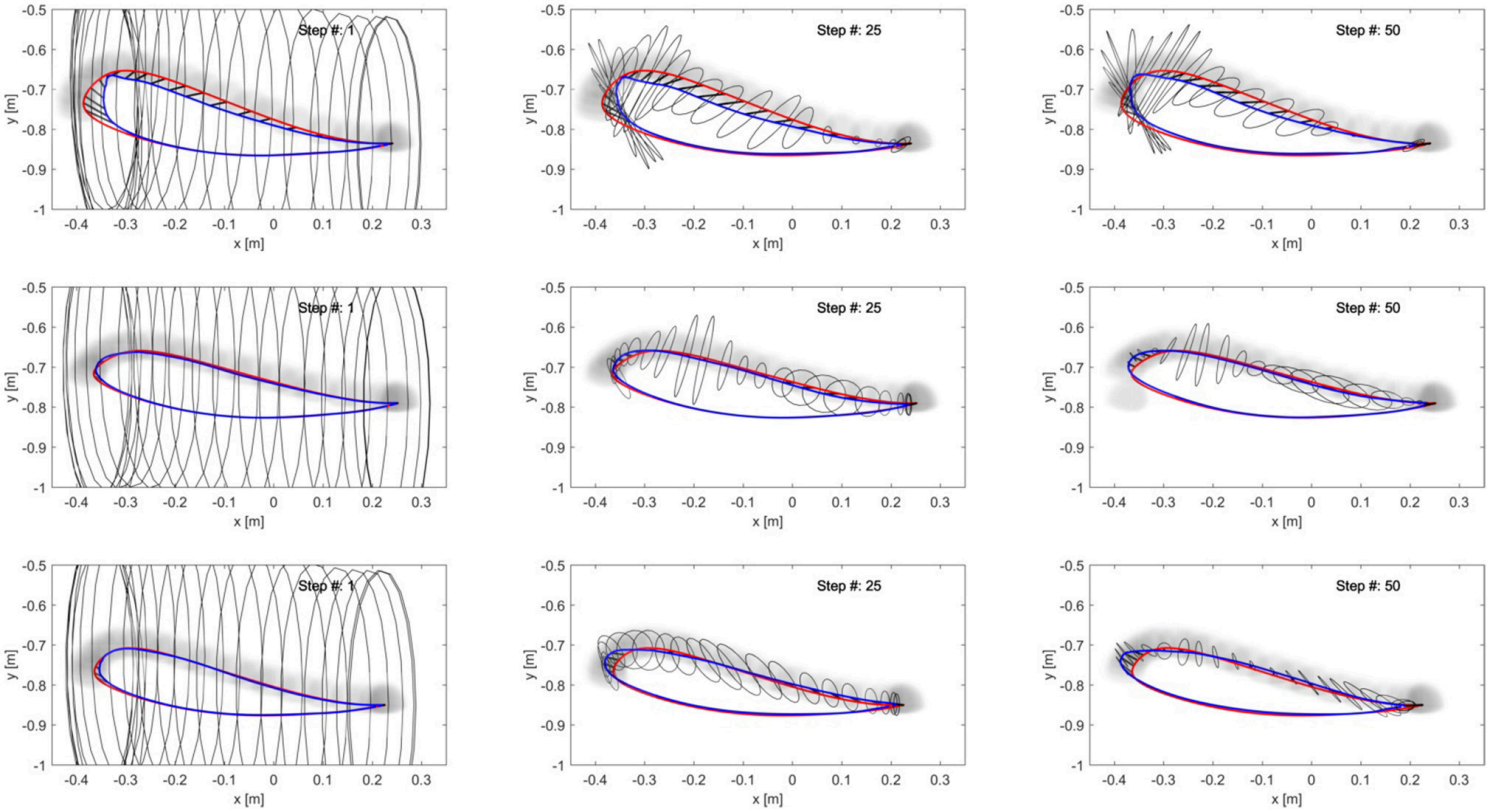
Adaptation of the end-point stiffness during the experiment for three different subjects. The initial stiffness ellipses are shown in the first column. To ensure a safe foot clearance during swing, the vertical stiffness maximum value was set higher than the horizontal stiffness. After 25 steps (2nd column) the stiffness ellipses are adapting to the error size and direction. At the last step of the adaptation (3rd column), the ellipses reached their final configuration. On the 1st line, data from the subject with SCI are shown: as expected, the final stiffness ellipses have a bigger size than those achieved by the able-bodied subjects in the 2nd and 3rd line.

Results (Figure 11) confirmed that the stiffness ellipses start from an initial size and shape (ratio major/minor axis = 5) and, based on Equation (13), subsequently adapt in shape, orientation and size to the errors at the ankle (Figure 12). During adaptation, the size of the stiffness ellipses adapts gradually to the kinematic error occurring in that gait phase. At every step, the orientation of the stiffness ellipses tends to align to the direction of the error in that gait window (Equation 13, second term).

**Figure 13:**
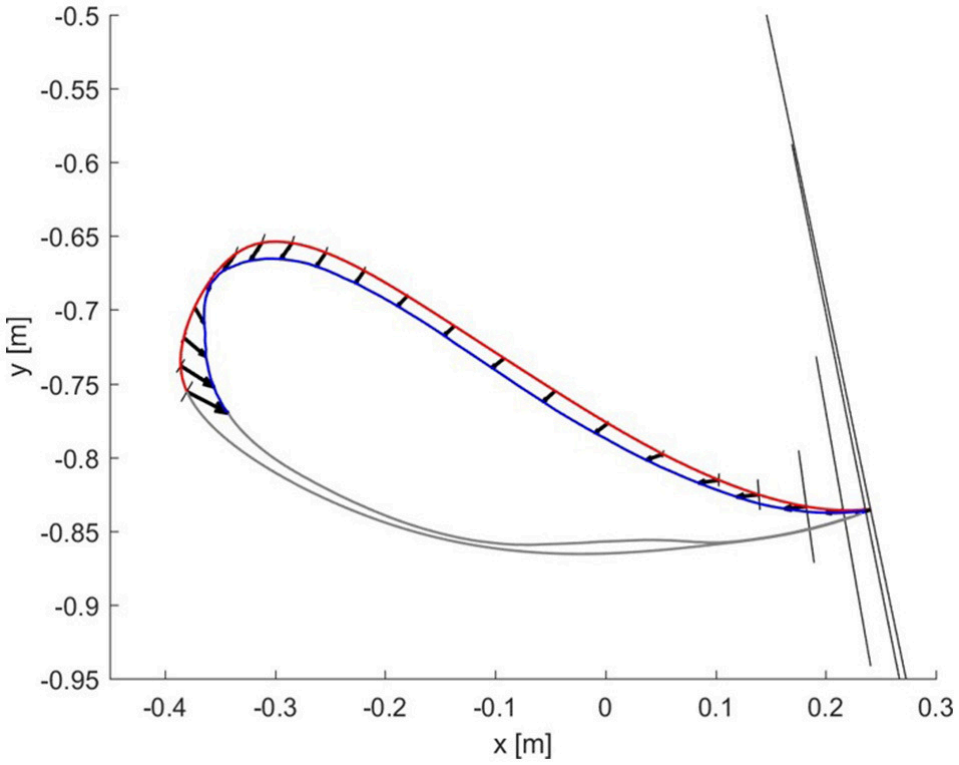
Resulting end-point stiffness ellipses caused by the joint controller in the subject with SCI. The resulting end-point stiffness is calculated from the hip and knee stiffness during the last step (50th) of the adaptation. The ellipses appear in the figure as lines since the minor axis is close to a null length. The kinematic error between the reference trajectory (red) and the actual trajectory (blue) of the ankle joint during swing phase is shown by the black vectors.

In contrast, Figure 13 shows the results for the joint controller, whereby hip and knee joint stiffness adapt separately (section Joint Space Formulation of an AAN Controller) and no coupling terms are present. Hence, the size, shape and orientation of the resulting end-point stiffness depend not only on the actual joint stiffness but also on the configuration of the leg segments (therefore, on the gait phase). It is clear that there is little or no correspondence between the errors performed in task space and the resulting end-point stiffness.

**Figure 14:**
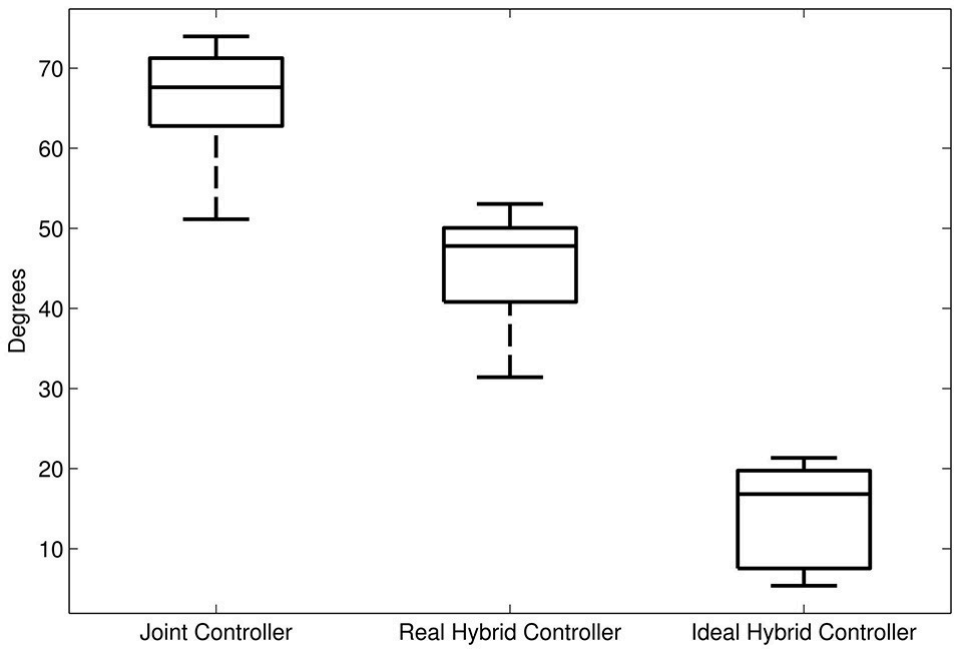
Average alignment (angle) between the major axis of the end-point stiffness ellipses and the direction of the error in the same gait window. First boxplot: alignment of the end-point stiffness ellipses resulting from the joint controller. Second boxplot: alignment of the end-point stiffness ellipses obtained in the experiments with the hybrid controller. Third boxplot: alignment of the end-point stiffness ellipses in the ideal case where the initial vertical stiffness of the hybrid control was set equal to the horizontal stiffness.

The alignment between the major axes of the ellipses and the error in the respective time window in the last 5 steps is greater (i.e., the angle is minimum) in the ideal hybrid controller (for K_xx_ = K_yy_) (Figure 14). The joint controller showed the worst performance in terms of alignment.

If we examine one of the critical points of the late swing phase, i.e., right before heel strike, in further detail (Figure 12), it becomes apparent that, especially in the subject with SCI, the hybrid controller generates an end-point stiffness ellipse rotated in the direction of the error (i.e., the stiffness is higher in the direction along which the error occurred). The adaptive joint controller (Figure 13) instead shows a very small stiffness value in that direction, but a high stiffness in a direction that does not apparently require any support.

## Discussion

The aim of our work was to develop an AAN controller for a lower limb exoskeleton which could optimally adapt the support based on the patient's ability to follow a reference trajectory. To achieve this, we examined and discussed the features and disadvantages of joint and end-point space formulation to control exoskeleton robots for the lower limbs. Then, we developed a proof-of-concept novel controller that combines the benefits of joint and end-point formulations: an adaptive hybrid joint/end-point space controller. We presented the results of a software simulation and, finally, the results of the tests on able-bodied subjects and one subject with SCI.

When developing a controller for gait exoskeletons, the choice of the formulation (joint or end-point) highly influences the apparent stiffness and damping rendered by the robot and it has an impact on how the reference trajectories (and safety features around them) are designed. While gait trajectories defined in joint space are closer to the hardware structure of the exoskeleton and similar to what gait analysis presents us, the trajectory of the foot during gait is a precise end-point control task (Winter, 1992). The human achieves certain trajectories in task-space thanks to the fact that the internal models take care of the proper muscle activations that guarantee the correct joint movements (Shadmehr and Mussa-Ivaldi, 1994). In both healthy and pathological conditions, different joint kinematic solutions are adopted to control the position and orientation of the end-point and, in particular, to achieve a safe trajectory of the foot during the swing phase (Winter, 1992). It has thus been hypothesized that the control of the foot trajectory during swing is a major focus of our central nervous system (CNS) during human locomotion (Ivanenko et al., 2003), as also supported by animal studies (Georgopoulos and Grillner, 1989). In contrast to trajectories defined in joint space, end-point trajectories of able-bodied subjects during swing show very little variability during walking on firm level ground and on the treadmill (Winter, 1992; Ivanenko et al., 2002; Awai and Curt, 2014). Instead, during stance phase the main task is not control of the foot trajectory, but rather the support and balance of the body weight. These functional tasks are accomplished by the control of hip, knee and ankle angles in a so called “support synergy” (Winter, 1995). The different tasks performed during swing and stance phase and the different models used for these two phases, support the use of the end-point formulation only during the swing phase of gait.

Depending on the formulation used in the controller, the resulting stiffness properties of the exoskeleton can vary significantly. This results in different magnitudes and directions of supportive torques. Considering the strengths and weaknesses of the joint and end-point formulation for impedance controllers, we proposed a hybrid joint/end-point controller in order to exploit the benefits of the end-point controller in shaping a desired end-point stiffness, while using an additional joint component to guarantee the correct angular trajectories of the joints. In previous research, the concept of a hybrid controller was introduced for an industrial manipulator that was programmed to follow a given end-point trajectory in the presence of external disturbances (both at the end-effector and at the joint level) (Smith et al., 2015). The torques calculated by the end-point controller were complemented with the torques obtained from a joint impedance controller only at those joints that were affected by large disturbance forces. This approach was proven more effective than either end-point or joint control alone to reduce the tracking error in the presence of perturbations at the end-effector and at the joint level.

The control over the end-point stiffness also opens new possibilities when developing a controller with assist-as-needed characteristics. The AAN implemented in end-point space can be directly programmed to adapt the magnitude and the direction of the stiffness based on the error of the subject in task space. Our experiments showed that the controller was capable of adapting the end-point stiffness based on the deviation of the subject from the foot reference trajectory. As expected, the application with a subject with SCI resulted in a higher final end-point stiffness than the able-bodied subjects. When comparing the alignment of the end-point stiffness ellipses generated by the different controllers, we saw that in the hybrid joint/end-point controller the stiffness was better aligned with the error direction. In this way, the controller directs the restoring forces in the direction where they are needed, thus providing a more “specific” support.

Emken et al. (2008) employed a similar approach on the end-effector robotic gait trainer ARTHuR. The end-point stiffness of the robot was adapted with an AAN algorithm that separately adapted horizontal and vertical stiffness. Our approach differs in that the end-point stiffness ellipses align to the direction where the maximum stiffness is required (i.e., the direction of the error). Interestingly, this behavior is close to the way humans adapt their stiffness in response to external disturbances: as shown in Burdet et al. (2001), the CNS can voluntarily control the magnitude, shape and orientation of the end-point stiffness in the upper limb. Moreover, several studies have found that the control of the foot trajectory is the major focus of our CNS during locomotion, both in the unimpaired (Winter, 1992; Ivanenko et al., 2002) and in the impaired spinal cord (Ivanenko et al., 2003; Awai and Curt, 2014). Therefore, a controller for robotic exoskeletons that is shaping the end-point position and the end-point stiffness can be considered as a “bioinspired” solution for the control of robotic devices for human interaction.

A further advantage of the adaptive end-point controller is that the error metric for the algorithm can be defined in task space. This allows us to consider explicitly the interaction between the foot and the environment and the spatio-temporal features of the foot trajectory. As Winter (1992) showed, foot clearance is sensitive to very small angular deviations in any of the joints of the lower limb kinematic chain. This means that, in order to guarantee a safe minimum toe clearance, one would have to design very restrictive deadbands in joint space, which would have a negative impact on freedom of movement for physiological deviations. In contrast, deadbands in task space can be designed to be restrictive only in the directions that are needed for safety, determining how much deviation can be tolerated in the vertical direction (crucial for avoiding stumbling) and in the horizontal direction, which corresponds to leading or lagging with respect to the reference trajectory. Moreover, with the end-point controller, it is possible to present the subjects with visual feedback on the errors in end-point space, which is much easier to process than feedback on joint position (Banala et al., 2009; Koopman et al., 2013; Krishnan et al., 2013), and use the same representation within the error metric of the adaptation algorithm. When used in the experiments with subjects, the adaptive hybrid joint/end-point controller required the use of an additional term to support against gravity: the vertical stiffness was set 5 times higher than the horizontal stiffness. Alternatively, an additional feed-forward term for compensating the effect of gravity could be added. Furthermore, lighter robots (e.g., LOPES; Veneman et al., 2007) would reduce the role of gravity and inertia of the system and thus the need to counteract them. The accuracy of the end-point position can be increased by adding a position sensor which measures directly the **x** coordinates of the ankle, instead of estimating them from the joint angles. While we derived necessary conditions for stability based on the approach proposed by Kronander and Billard (2016) and we took several precautions to guarantee safety, the stability of the AAN hybrid controller is something that requires further investigation.

The single subject with SCI with whom we tested the adaptive and hybrid end-point/joint controller did not show abnormal muscle activation synergies. Extra care should be taken when using the hybrid control with patients that present abnormal synergies or other strong compensatory movements. There might be cases where, despite an almost physiological end-point trajectory, hip and knee angles remain anomalous (Awai and Curt, 2014). In such cases the hybrid control should be extended by a term that counteracts joint position deviations, as in the approach proposed by Smith et al. (2015), where a joint impedance term was added only when large disturbances at the joint level were detected. Before drawing any conclusions on the benefits of this novel controller in treating subjects with gait disabilities, more tests are needed to study how the controller would react to different impairments such as spasticity.

As a future step, the application of our adaptive hybrid joint/end-point controller concept to other rehabilitation robots, e.g., upper limb exoskeletons [such as the ARMin (Nef et al., 2007), Armeo®Power (Hocoma AG) or ALEx (Pirondini et al., 2014)] would be of great interest, because a vast body of literature has investigated how humans adapt their upper-limb stiffness based on the task and on external disturbances (Shadmehr, 1993; Burdet et al., 2001) and it would be instructive to use an adaptive controller similar to the one presented in this work to test its interaction with the human arm.

## Conclusion

The adaptive controller presented in this paper implements our ideas of a safe controller combining an end-point impedance controller with a joint damping controller into a “hybrid” joint/end-point controller. The controller was tested successfully with able-bodied human subjects and one subject with spinal cord injury. With this approach, it was possible to implement an adaptive controller that shapes the end-point stiffness according to the direction and the magnitude of the error performed at the ankle. In contrast to other applications, the hybrid controller adapts the end-point stiffness to selectively counteract certain errors while leaving the robot compliant in other directions. The adaptive controller proposed in this paper is a patient-cooperative, bio-inspired solution for more human-oriented rehabilitation robots, which fulfills the requirement of “adaptability” identified by many studies in the field of rehabilitation robotics (Iosa et al., 2016) and may be used on other devices, including upper extremity rehabilitation robots.

## Author contributions

SM contributed to the development of the controller described in this work and to the study design, performed the experiments, analyzed the data and wrote the manuscript draft. NR developed the controller described, performed the experiments, analyzed the data and revised the manuscript. LL provided significant advice during the whole study and revised the manuscript. AM-C conceived the study and the controller, supported significantly in the development of the controller, interpretation of the data, and contributed to the manuscript draft. All authors contributed to manuscript revision, read and approved the submitted version.

### Conflict of interest statement

SM, LL, and AM-C at the time of the study worked at Hocoma AG in the R&D Department. SM, NR, and LL currently work at Hocoma AG.

## Abbreviations

AAN: Assist-as-needed
PD: Proportional Derivative control
CNS: Central Nervous System
CoR: Center of Rotation
ROM: Range of Motion
SCI: Spinal Cord Injury.
