# Supplementary material for: An Adaptive and Hybrid End-Point/Joint Impedance Controller for Lower Limb Exoskeletons
Source: Front Robot AI. 2018 Oct 22;5:104. doi: 10.3389/frobt.2018.00104 (PMC7805861; doi:10.3389/frobt.2018.00104)
Supplement: Supplementary file 1 [file Data_Sheet_1.pdf]

1 Appendix: Relationship between joint and end-point spaces

1.1 Forward kinematics

In this paper we examine the behavior of two controllers applied to a two-segment robotic device with two actuated joints, which correspond to the hip and knee joint of the human subject.

In the paper we referred to joint angular trajectories, **q**:

$$\mathbf{q} = \begin{bmatrix} q_{hip} \\ q_{knee} \end{bmatrix} \tag{A.1}$$

We calculate the position of the end-point of the two-segment kinematic chain in Cartesian space, using the sign convention shown in **Figure 1**:

$$\mathbf{x} = \begin{bmatrix} l_1 \sin(q_{hip}) + l_2 \sin(q_{hip} + q_{knee}) \\ -l_1 \cos(q_{hip}) - l_2 \cos(q_{hip} + q_{knee}) \end{bmatrix} = \begin{bmatrix} x \\ y \end{bmatrix} \tag{A.2}$$

1.2 Transformations between end-point forces and joint torques

In our system, a set of commanded joint torques, $\boldsymbol{\tau}$, are used to control the robotic joints:

$$\boldsymbol{\tau} = \begin{bmatrix} \tau_{hip} \\ \tau_{knee} \end{bmatrix} \tag{A.3}$$

We can obtain the resulting forces applied at the end-point, $\boldsymbol{F}_x$, in correspondence of a given set of applied joint torques, through this transformation:

$$\mathbf{F}_x = (\mathbf{J}[\boldsymbol{q}]^T)^{-1}\boldsymbol{\tau}_q \tag{A.4}$$

where $\mathbf{J}[\mathbf{q}]$ is the Jacobian matrix, that represents the differential relationship between the joint displacements and the resulting end-point motion (i.e. the sensitivity of each individual end-

point coordinate to individual joint displacement). The Jacobian matrix is defined for each set of joint coordinates. The Jacobian matrix for our system can be calculated as follows:

$$\boldsymbol{J}(\boldsymbol{q}_{act}) = \begin{bmatrix} l_1\cos(q_{hip}) + l_2\cos(q_{hip}+q_{knee}) & l_2\cos(q_{hip}+q_{knee}) \\ l_1\sin(q_{hip}) + l_2\sin(q_{hip}+q_{knee}) & l_2\sin(q_{hip}+q_{knee}) \end{bmatrix} \quad \text{(A.5)}$$

On the other side, to obtain the joint torques necessary to provide the desired end-point forces, we can use the inverse of Eq. A.4:

$$\boldsymbol{\tau}_q = \boldsymbol{J}[\boldsymbol{q}]^T \mathbf{F}_x \quad \text{(A.6)}$$

1.3 Transformations between joint stiffness and end-point stiffness

For each set point along the angular reference trajectory, we want to study the relationship between torque and angular displacement; this relationship is represented by the joint stiffness matrix $\boldsymbol{K}_j$ (Shadmehr, 1993):

$$\boldsymbol{K}_j = \frac{d\boldsymbol{\tau}}{d\boldsymbol{q}} \quad \text{(A.7)}$$

Therefore, for small angular displacement around the set point we can calculate the desired restoring torques:

$$\begin{bmatrix} \tau_{hip} \\ \tau_{knee} \end{bmatrix} = \mathbf{K}_j \begin{bmatrix} dq_{hip} \\ dq_{knee} \end{bmatrix} = \begin{bmatrix} K_{hh} & K_{hk} \\ K_{kh} & K_{kk} \end{bmatrix} \begin{bmatrix} dq_{hip} \\ dq_{knee} \end{bmatrix} \quad \text{(A.8)}$$

In a similar way, in task space, the end-point stiffness matrix $\mathbf{K}_x$ represents the ratio between linear force and linear displacement:

$$\mathbf{K}_x = \frac{d\boldsymbol{F}}{d\boldsymbol{x}} \quad \text{(A.9)}$$

The desired restoring forces can be calculated knowing the end-point stiffness matrix:

$$\begin{bmatrix} F_x \\ F_y \end{bmatrix} = \mathbf{K}_x \begin{bmatrix} dx \\ dy \end{bmatrix} = \begin{bmatrix} K_{xx} & K_{xy} \\ K_{yx} & K_{yy} \end{bmatrix} \begin{bmatrix} dx \\ dy \end{bmatrix} \tag{A.10}$$

The joint and end-point stiffness matrices are symmetric (Shadmehr, 1993), therefore $K_{xy} = K_{yx}$ and $K_{hk} = K_{kh}$.

To calculate the perceived stiffness $\mathbf{K}_x$ at the end-point we can apply this calculation to the joint stiffness $\mathbf{K}_j$:

$$\mathbf{K}_x = (\mathbf{J}[\boldsymbol{q}]^{\mathrm{T}})^{-1}\mathbf{K}_q(\mathbf{J}[\boldsymbol{q}])^{-1} \tag{A.11}$$

1.4 Stiffness ellipses

As shown in (Mussa-Ivaldi et al., 1985), if we take d**x** as a unitary vector with a direction that changes gradually from 0° to 360° and we multiply it for $\mathbf{K}_x$, we obtain the corresponding output force vectors. They describe an ellipse, where the major axis indicates the direction along which the stiffness is higher, whereas the minor axis is the direction of minimum stiffness. These directions are the eigenvectors of the stiffness matrix and the magnitude of the major and minor axes are its eigenvalues.

We can also visually represent the stiffness around a set point using force and torque fields in end-point and joint space, respectively. For small displacements around a set point, we can plot the restoring forces or torques as shown above (Equations A.8 and A.10). It is interesting to notice that, except for the directions of the major and minor axes of the ellipse, the restoring forces and torques are not co-linear with the correspondent displacement vectors.

2 Appendix: Necessary conditions for stability

Given the non-linear and adaptive nature of the human-robot interaction controller presented in this paper, deriving necessary and sufficient conditions for stability can be a daunting task. However, previous work in adaptive impedance controllers and human-robot interaction

(Kronander and Billard, 2016; Smith et al., 2015; Yang et al., 2011), can serve as a starting point to define, at the very least, necessary (although not sufficient) conditions for stability. These conditions, combined with implementation of safety mechanisms (software and hardware), minimize the risk of undesired exoskeleton behaviors which might compromise the safety of use.

The dynamics of the system during swing can be expressed as:

$$\mathbf{H}[\mathbf{q}_{act}]\,\ddot{\mathbf{q}}_{act} + \mathbf{C}[\mathbf{q}_{act},\ \dot{\mathbf{q}}_{act}]\,\dot{\mathbf{q}}_{act} + \mathbf{G}[\mathbf{q}_{act}] = \boldsymbol{\tau}_{swing} + \boldsymbol{\tau}_{e} \tag{A.12}$$

where $\boldsymbol{\tau}_{swing}$ is the controller torque in Eq. 7 and $\boldsymbol{\tau}_e$ is the external torque, which can be caused by disturbances or interactions with the user. In our approach, we used the concept of generalized elasticities (Vallery et al., 2009) to compensate for the system dynamics ($\boldsymbol{\tau}_{comp}$ in Eq. 7). The compensation torque can be approximated as:

$$\boldsymbol{\tau}_{comp} = \boldsymbol{\eta}\left[\mathbf{q}_{act}, \dot{\mathbf{q}}_{act}, \ddot{q}_{ref}\right] \approx \mathbf{H}\,\ddot{\mathbf{q}}_{ref} + \mathbf{C}\,\dot{\mathbf{q}}_{act} + \mathbf{G} \tag{A.13}$$

The closed-loop system dynamics become:

$$\mathbf{H}\left(\ddot{\mathbf{q}}_{act} - \ddot{\mathbf{q}}_{ref}\right) = \boldsymbol{\tau}_{xPD} + \boldsymbol{\tau}_{qD} + \boldsymbol{\tau}_{e} \tag{A.14}$$

Substituting Eq. 10 in A.14, the system becomes:

$$\mathbf{H}\,\ddot{\mathbf{e}} + \mathbf{B}_{tot}\,\dot{\mathbf{e}} + \mathbf{K}_{tot}\,\mathbf{e} = \boldsymbol{\tau}_{e} \tag{A.15}$$

Where $\mathbf{H}$ is the symmetric and positive definite inertia matrix; and $\mathbf{K}_{tot}$ and $\mathbf{B}_{tot}$ are symmetric matrices that represents the overall time-varying stiffness and damping terms in joint space.

In (Kronander and Billard, 2016), the authors prove that the system in Eq. A.15 with $\boldsymbol{\tau}_e = 0$ is globally uniformly stable if there exists an $\alpha > 0$, such that $\forall t \geq 0$:

1) $\alpha\mathbf{H} - \mathbf{B}_{tot}$ is negative semidefinite

2) $\dot{\mathbf{K}}_{tot} + \alpha\dot{\mathbf{B}}_{tot} - 2\alpha\mathbf{K}_{tot}$ is negative semidefinite

Note, however, that the condition for stability defined in (Kronander and Billard, 2016) assumes that $\mathbf{H}$ is constant. In our case, the inertia matrix changes over time as the leg swings.

To extend the concept suggested by (Kronander and Billard, 2016) to include a time-varying inertia, we can use the same procedure and derive the conditions for stability using the following Lyapunov candidate function:

$$V[\mathbf{e},\dot{\mathbf{e}};t] = \frac{1}{2}(\dot{\mathbf{e}} + \alpha\mathbf{e})^{\mathrm{T}}\, \mathbf{H}[t]\, (\dot{\mathbf{e}} + \alpha\mathbf{e}) + \frac{1}{2}\mathbf{e}^{\mathrm{T}}\, \mathbf{\Psi}[t]\, \mathbf{e} \tag{A.16}$$

The derivative (in compact notation) becomes:

$$\dot{V} = (\dot{\mathbf{e}} + \alpha\mathbf{e})^{\mathrm{T}}\, \mathbf{H}\, (\ddot{\mathbf{e}} + \alpha\dot{\mathbf{e}}) + \frac{1}{2}(\dot{\mathbf{e}} + \alpha\mathbf{e})^{\mathrm{T}}\, \dot{\mathbf{H}}\, (\dot{\mathbf{e}} + \alpha\mathbf{e}) + \mathbf{e}^{\mathrm{T}}\, \mathbf{\Psi}\, \dot{\mathbf{e}} + \frac{1}{2}\mathbf{e}^{\mathrm{T}}\, \dot{\mathbf{\Psi}}\, \mathbf{e} \tag{A.17}$$

Substituting Eq. A.15 in Eq. A.17, results:

$$\dot{V} = \dot{\mathbf{e}}^{\mathrm{T}} \left(\alpha\mathbf{H} + \frac{1}{2}\dot{\mathbf{H}} - \mathbf{B}\right)\dot{\mathbf{e}} + \dot{\mathbf{e}}^{\mathrm{T}} \left(\alpha\dot{\mathbf{H}} - \mathbf{K} - \alpha\mathbf{B} + \alpha^2\mathbf{H} + \mathbf{\Psi}\right)\mathbf{e} + \mathbf{e}^{\mathrm{T}} \left(\frac{1}{2}\dot{\mathbf{\Psi}} - \alpha\mathbf{K} + \frac{\alpha^2}{2}\dot{\mathbf{H}}\right) \mathbf{e} \tag{A.18}$$

Similar to (Kronander and Billard, 2016), we get rid of the cross term by proposing:

$$\mathbf{\Psi} = \mathbf{K} + \alpha\mathbf{B} - \alpha^2\mathbf{H} - \alpha\dot{\mathbf{H}} \tag{A.19}$$

Eq. A.18 then becomes:

$$\dot{V} = \dot{\mathbf{e}}^{\mathrm{T}} \left(\alpha\mathbf{H} + \frac{1}{2}\dot{\mathbf{H}} - \mathbf{B}\right)\dot{\mathbf{e}} + \mathbf{e}^{\mathrm{T}} \left(\frac{1}{2}\dot{\mathbf{K}} + \frac{\alpha}{2}\mathbf{B} - \alpha\dot{\mathbf{K}} - \frac{\alpha}{2}\ddot{\mathbf{H}}\right)\mathbf{e} \tag{A.20}$$

Thus, the system with time-varying inertia is globally uniformly stable if there exists an $\alpha > 0$, such that $\forall t \geq 0$:

Condition 1: $\alpha\mathbf{H} + \frac{1}{2}\dot{\mathbf{H}} - \mathbf{B}_{tot}$ is negative semidefinite (A.21)

Condition 2: $\dot{\mathbf{K}}_{tot} + \alpha\dot{\mathbf{B}}_{tot} - 2\alpha\mathbf{K}_{tot} - \alpha\ddot{\mathbf{H}}$ is negative semidefinite (A.22)

Note that, while this derivation includes a time-varying inertia, stiffness and damping, it does not take into account that these time-varying matrices are configuration-dependent. In our case, the three matrices $\mathbf{H}$, $\mathbf{K}_{tot}$ and $\mathbf{B}_{tot}$ depend on the configuration angles $\mathbf{q}_{act}$. However, to some degree, the conditions for stability in A.21 and A.22 can provide some necessary boundaries to the different parameters.

From *Condition 2*, one can notice that $\mathbf{K}_{tot}$ must be always positive semidefinite if the system is to be globally stable at a given static kinematic configuration (i.e. $\ddot{\mathbf{H}} = \mathbf{0}$) and a constant stiffness matrix. For a 2x2 matrix $\mathbf{K}_{tot}$, this condition can be easily implemented in software by constraining the values of the matrix $\mathbf{K}_{tot}$ such that $\forall t$:

$$K_{11} \geq 0 \;\;\&\;\; K_{22} \geq 0 \;\;\&\;\; -\sqrt{K_{11}K_{22}} \leq K_{12} \leq \sqrt{K_{11}K_{22}} \;\;\&\;\; K_{21} = K_{12} \qquad \text{(A.23)}$$

Under some assumptions on walking parameters, one can calculate the values of $\ddot{\mathbf{H}}$ along different expected trajectories (based on pre-defined walking speed, cadence, etc.). Assuming constant stiffness and damping matrices, the term $2\mathbf{K}_{tot} + \ddot{\mathbf{H}}$ must remain positive semidefinite along all those possible trajectories, thus:

$$K_{11} \geq -\frac{1}{2}\ddot{H}_{11} \;\;\&\;\; K_{22} \geq -\frac{1}{2}\ddot{H}_{22} \;\;\&\;\; -\frac{1}{2}\ddot{H}_{12} - \upsilon_K \leq K_{12} \leq -\frac{1}{2}\ddot{H}_{12} + \upsilon_K \qquad \text{(A.24)}$$

where

$$\upsilon_K = \frac{1}{2}\sqrt{\ddot{H}_{11}\ddot{H}_{22} + 2K_{11}\,\ddot{H}_{22} + 2K_{22}\,\ddot{H}_{11} + 4K_{11}K_{22}} \qquad \text{(A.25)}$$

Conditions A.23 and A.24 are necessary, but not sufficient conditions, for $\mathbf{K}_{tot}$ as long as the stiffness and damping remain constant or decrease.

More generally, when stiffness and damping matrices increase, one could verify stability by following the methodology in (Kronander and Billard, 2016), such that $\dot{\mathbf{K}}_{tot} + \alpha\dot{\mathbf{B}}_{tot} - 2\alpha\mathbf{K}_{tot} - \alpha\ddot{\mathbf{H}}$ remains negative semidefinite. It follows:

$$\overline{\lambda}(\dot{\mathbf{K}}) < \alpha\left(2\underline{\lambda}(\mathbf{K}) + \underline{\lambda}(\ddot{\mathbf{H}}) - \overline{\lambda}(\dot{\mathbf{B}})\right) \tag{A.26}$$

where $\overline{\lambda}(\cdot)$ and $\underline{\lambda}(\cdot)$ denote the largest and smallest eigenvalues.

Similarly, one could find the limits for $\mathbf{B}_{tot}$ so that $\alpha\mathbf{H} + \frac{1}{2}\dot{\mathbf{H}} - \mathbf{B}_{tot}$ remains negative semidefinite for a given $\alpha > 0$:

$$B_{11} \geq \frac{1}{2}\left(\ddot{H}_{11} + 2\alpha H_{11}\right) \ \& \ B_{22} \geq \frac{1}{2}\left(\ddot{H}_{22} + 2\alpha H_{22}\right) \ \&$$

$$\frac{1}{2}\left(\ddot{H}_{12} + 2\alpha H_{12}\right) - \upsilon_B \leq B_{12} \leq \frac{1}{2}\left(\ddot{H}_{12} + 2\alpha H_{12}\right) + \upsilon_B \tag{A.27}$$

where

$$\upsilon_B = \frac{1}{2}\sqrt{-\left(2B_{11} - \dot{H}_{11} - 2\alpha H_{11}\right)\left(2B_{22} + \dot{H}_{22} - 2\alpha H_{22}\right)} \tag{A.28}$$

More generally:

$$\underline{\lambda}(\mathbf{B}) > \frac{1}{2}\overline{\lambda}(\dot{\mathbf{H}}) + \alpha\overline{\lambda}(\mathbf{H}) \tag{A.29}$$

Thus, the least conservative value for α is by:

$$\alpha = \min_{t} \frac{\underline{\lambda}(\mathbf{B}) - \frac{1}{2}\overline{\lambda}(\dot{\mathbf{H}})}{\overline{\lambda}(\mathbf{H})} \tag{A.30}$$

As stated earlier, these conditions are necessary, but not sufficient, to guarantee stability of the complex interactions of both the human and robot adapting simultaneously with our proposed controller. Thus, we highly recommend that the implementation of such adaptive controllers is

accompanied by thorough safety mechanisms, such as limits on the current provided to the motors and on its rate of change, saturation values, virtual walls etc. to fully minimize the risk of the controller getting unstable.

References

Kronander, K., and Billard, A. (2016). Stability Considerations for Variable Impedance Control. *IEEE Trans. Robot.* 32, 1298–1305. doi:10.1109/TRO.2016.2593492.

Mussa-Ivaldi, F., Hogan, N., and Bizzi, E. (1985). Neural, mechanical, and geometric factors subserving arm posture in humans. *J. Neurosci.* 5, 2732–2743. doi:10.1056/NEJM197706162962404.

Shadmehr, R. (1993). Control of Equilibrium Position and Stiffness Through Postural Modules. *J. Mot. Behav.* 25, 228–241. doi:10.1080/00222895.1993.9942052.

Smith, A., Yang, C., Ma, H., Culverhouse, P., Cangelosi, A., and Burdet, E. (2015). Novel hybrid adaptive controller for manipulation in complex perturbation environments. *PLoS One* 10, e0129281. doi:10.1371/journal.pone.0129281.

Vallery, H., Duschau-Wicke, A., and Riener, R. (2009). Optimized passive dynamics improve transparency of haptic devices. in *Proceedings - IEEE International Conference on Robotics and Automation*, 301–306. Available at: http://www.scopus.com/scopus/record/display.url?fedsrfIntegrator=MEKPAPERS-SCOCIT&origin=fedsrf&view=basic&eid=2-s2.0-70350349707.

Yang, C., Ganesh, G., Haddadin, S., Parusel, S., Albu-Schaeffer, A., and Burdet, E. (2011). Human-Like Adaptation of Force and Impedance in Stable and Unstable Interactions. *Robot. IEEE Trans.* 27, 918–930. doi:10.1109/TRO.2011.2158251.
